# Age, muscle, and gender specific characterization of muscle degeneration in a mouse model of calpainopathy

**DOI:** 10.1038/s41598-025-17742-3

**Published:** 2025-09-12

**Authors:** Nicolina Südkamp, Jacqueline Heinen-Weiler, Marlena Rohm, Michaela Zaik, Nassam Daya, Anne-Katrin Güttsches, Carsten Theiss, Andreas Roos, Tobias Ruck, Frank Jacobsen, Lara Schlaffke, Matthias Vorgerd

**Affiliations:** 1 Department of Neurology, BG-University Hospital Bergmannsheil gGmbH, Ruhr University Bochum, Bochum, Germany; 2 Heimer Institute for Muscle Research, BG-University Hospital Bergmannsheil gGmbH, Bochum, Germany; 3 Medical Imaging Center (MIC), Electron Microscopy Medical Analysis – Core Facility (EMMACF), Faculty of Medicine, Ruhr University Bochum, 44780 Bochum, Germany; 4 Department of Cytology, Ruhr University Bochum, 44780 Bochum, Germany; 5 Department of Pediatric Neurology, Centre for Neuromuscular Disorders, Centre for Translational Neuro- and Behavioral Sciences, University Duisburg-Essen, Essen, Germany; 6 Brain and Mind Research Institute, Children’s Hospital of Eastern Ontario Research Institute, Ottawa, ON Canada; 7 Department of Neurology, Medical Faculty and University Hospital Düsseldorf, Heinrich Heine University, Düsseldorf, Germany; 8 Department Medical Engineering, FH Dortmund, University of Applied Sciences and Arts, Dortmund, Germany

**Keywords:** Calpainopathy, Calpain 3, Mouse model, Histopathology, Neurology, Neurological disorders, Neuromuscular disease

## Abstract

Establishing well-described mouse models of hereditary diseases is increasingly important for testing new therapeutic approaches, such as gene replacement therapy. In particular, a detailed description of muscle pathology, especially at early timepoints of the disease, is crucial for determining the optimal timepoint for drug delivery and evaluation of therapeutic success. In this study, we aimed to characterize and quantify the muscle pathology and myofibre morphology of different muscles in a new mouse model for calpainopathy as an example of the heterogenous group of limb girdle muscular dystrophies compared to wildtype controls during the disease. We analysed motor function and muscle tissue of wildtype and *Capn3-*transgenic mice per gender from 1.5 to 15 months of age. While transgenic mice did not develop restrictions in motor function, tested with grip strength measurement, beam walk and four limb wire hanging test, during this period, gastrocnemius, soleus and psoas muscles showed progressive histopathological and ultrastructural changes. Importantly, we also detected gender-specific differences in general muscle structure and in muscle pathology in the mouse model of calpainopathy. We developed a score to classify pathology of muscles in this mouse model using percentage of myocytes with centralized nuclei to increase objectivity and comparability when using this mouse model.

**Supplementary Information:**

The online version contains supplementary material available at 10.1038/s41598-025-17742-3.

## Introduction

Calpainopathy, or limb girdle muscular dystrophy (LGMD) type 2A/R1 is the most common subtype of all LGMD forms (about 30%)^1–3^. With an overall prevalence of 1–9:100.000^3^, LGMD belongs to the group of rare diseases. LGMD encompasses a group of autosomal dominant and recessive inherited disorders with clinical presentation of progressive proximal muscle weakness and atrophy^3,4^. The clinical manifestation of symptoms varies depending on the type of mutation, gender and other unidentified factors^1,5^. Calpainopathy is inherited in an autosomal recessive manner, whereby more than 450 different pathogenic variants are known to date. The calpain 3-gene (*Capn3*) on chromosome 15 is affected^1,6,7^. All known mutations result in a loss of function of calpain 3 (CAPN3). The protein CAPN3 acts as an intracellular calcium-dependent cysteine protease presenting with many different functions and associated protein–protein interactions within skeletal muscle cells^6,8,9^. Key functions include regulation of apoptosis, muscle cell differentiation, formation of sarcomeres and regulation of the cytoskeleton^6,8,9^. CAPN3 is localized to the contractile apparatus and is there stabilized by Titin^10^.

Clinically, the definitive diagnosis of calpainopathy is frequently made several years after symptom onset and is often guided by muscle pathology findings including Western blot analysis to validate missing or reduced amounts of CAPN3 in muscle tissue. However, the diagnostic gold standard is genetic testing based on the clinical suspicion of muscular dystrophy^11–13^. Histologically, necrosis of affected muscle fibres, as well as muscle fibre regeneration, high variability of fibre diameter and fibrosis can be found^2,6,14^.

Overall, muscle biopsies represent only a small cross-section of one particular muscle at one time point within a chronic and progressive disease. Calpainopathy is also known to affect many muscles but with varying severity^15^. Due to the limited amount of tissue and the invasive technique, human tissue is not sufficient to study the exact histopathological features and time course of the disease. Animal models for calpainopathy have been used for several years, including different mouse models^16–19^ and lately also a zebrafish model^20,21^. These animal models are heterogeneous and not available to the scientific community. In consideration of emerging therapeutic approaches, the use of a consistent and well described mouse model of calpainopathy is of great importance.

Recently, a mouse strain with a mutant of the *Capn3* gene, which carries a 1759 nucleotide deletion generated by CRISPR/Cas9 endonuclease-mediated genome editing, has been designed (JAX stock #031211) and is available from a public mouse repository. Currently, no detailed information about the muscle pathology is available for this strain. However, this information is essential for drug testing targeting new therapeutic approaches, especially at early time points of the disease. Notably, this myopathological knowledge is crucial for determining the optimal timepoint for drug delivery and would enable a robust evaluation of therapeutic success.

To systematically address this gap of knowledge, in this descriptive study, we aimed to characterize the phenotype and the muscle pathology by analysing different muscles in this new mouse model for calpainopathy compared to wildtype controls. We analysed muscle tissue of wildtype (WT) and *Capn3-*mutant mice (JAX stock #031211) of both genders at the age of 1.5 months, 3 months, 5 months, 10 months and 15 months. As no characterization is available at the producer, chosen timepoints were adapted to studies using other mouse models of calpainopathy^16–18^. Expression of *Capn3* specific mRNA was investigated via quantitative PCR in addition to studies of CAPN3 protein abundance determined by Western blot. Muscle tissue was examined for ultra-structural alterations using transmission electron microscopy (TEM). Furthermore, histopathologic features were systematically quantified, and a simplified scoring system for assessing muscle pathology in this mouse model was developed to facilitate easier and more consistent monitoring of the severity of muscle pathology in this mouse model.

## Material and methods

### Animals

In this study, 129S4/SvJaeJ-*Capn3*^*em5Lutzy*^/J mice were used as an animal model for calpainopathy (JAX stock #031211, referred to as Capn3-deficient) and 129S4/SvJaeJ mice (JAX stock #009104) as controls (referred to as WT). Plasmids encoding a single guide RNA designed to mediate a 1759-nucleotide deletion within the *Capn3* locus, along with a plasmid expressing the *Cas9* endonuclease, were microinjected into the cytoplasm of fertilized oocytes derived from the 129S4/SvJaeJ strain, selected at the pronuclear stage. Embryos confirmed to be correctly edited were transferred into the uteri of pseudopregnant recipient females. The mutation leads to a deletion of exon 2 and 3 of the *Capn3* gene. Mice were housed in custom-made ventilated and acclimatized holding cupboards in a rodent-housing room (12-h light/dark cycle) with unlimited access to food and water. Experiments were carried out in accordance with the European Communities Council Directive of September 22nd, 2010 (2010/63/EU) for care of laboratory animals and were conducted according to the guidelines of the German Animal Protection Law. Experiments were authorized in advance by the North Rhine-Westphalia (NRW) State Authority (Landesamt für Arbeitsschutz, Naturschutz, Umweltschutz und Verbraucherschutz, NRW). Three to four male and female mice per genotype were sacrificed by cervical dislocation after anaesthesia with 3% isoflurane at the age of 1.5 months, 3 months, 5 months, 10 months and 15 months. A power analysis was carried out to calculate the required sample size in advance. In accordance with the 3R-concept, all efforts were made to minimize the number of animals used. All experiments were performed, and the study is reported in accordance with the ARRIVE guidelines.

### In vivo phenotyping

For in vivo phenotyping, standard and easily accessible methods with only minimal burden to mice have been used. Body weight of mice was tracked at the previously mentioned time points. Grip strength of the forelimb was measured using the Grip Strength Meter V2.5.1 (TSE Systems, Germany). The measurement was repeated ten times, and the average of the three best attempts was calculated and normalized to body weight at the respective time point. For the beam walk test, a beam of one meter length and 8 mm width was used, resting 30 cm above a build-up cage filled with 5–7 cm of litter to prevent injuries in case of falls. A dark box with nesting material from the home cages was placed at the end of the beam. After training sessions, the time to cross the central 80 cm was recorded at each time point, and the number of slips was counted. For the four limb wire hanging test, mice were placed on a wire, which was then flipped upside down and placed 30 cm above a build-up cage filled with 5–7 cm of litter to prevent injuries. At each time point, the latency to fall was measured until a total hanging time of 180 s. Each mouse had three attempts with a resting period of one minute in between.

### Sample preparation

After sacrificing the mice as described above, psoas muscle, soleus and gastrocnemius muscle of both hindlimbs were dissected and snap-frozen in liquid-nitrogen-cooled isopentane and kept at − 80 °C until use. Cryosections were prepared using a microtome (Leica, Mannheim, Germany). Muscle specimens were chosen based on results in other mouse models of calpainopathy^16,17^ to enhance comparability. Additionally, quadriceps muscle and diaphragm were also dissected and examined (see Supplementary Figure S1).

### RNA isolation, cDNA synthesis and real-time qPCR

For isolation of RNA, 20 cryosections of 10 µm thickness of soleus and gastrocnemius muscle were prepared using an endoribonuclease-free environment and total RNA was isolated using the Quick-RNA™ MiniPrep Kit (Zymo Research, Irvine, CA, USA) with a DNase digestion step following the manufacturer’s protocol. For spectrometric determination of RNA concentration, 1 µl of the samples was measured using a biophotometer (Eppendorf SE, Hamburg, Germany) with a wavelength of 260 nm. Purity of RNA was checked by the ratio OD260/OD280. Equal amounts of RNA (50 ng) of each sample were reverse-transcribed into cDNA using the High-Capacity cDNA Reverse Transcription Kit (Applied Biosystems by Thermo Fisher Scientific Inc., Waltham, Massachusetts, USA) according to the manufacturer’s protocol. Equal amounts of cDNA were used to measure expression levels of *Capn3* using specific primers (5′-ACAACAATCAgCTggTTTTCACC- 3′, 5′-CAAAAAACTCTgTCACCCCTCC-3′). 18SrRNA was used as a housekeeping gene (primers: 5′-CACAgTTATCCAAgTAggAgAgg-3′, 5′-gAAACTgCgAATggCTCATTAAA-3′). Real-time qPCR was performed using iTaq Universal SYBR® Green Supermix (Bio-Rad, Feldkirchen, Germany) according to the manufacturer’s instructions in a CFX96 Touch Real-Time PCR Detection System light cycler (Bio-Rad, Feldkirchen, Germany). The relative expression of *Capn3* to *18S* was calculated using CFX Maestro™ Software (Bio-Rad, Feldkirchen, Germany).

### Western blot

For western blot of CAPN3, 20 cryosections of 10 µm thickness of soleus and gastrocnemius muscle were prepared and homogenized by vortexing in ice-cold lysis buffer containing 4 mol/L urea, 20 mmol/L Tris–HCL (pH 8,5), 5 mmol/L DTT, 1.5 mmol/L MgCl_2_, 1% Triton-X-100, 25 U/ml Benzonase and 2 µmol/L protease-inhibitor E64. Protein concentration was determined using the RC/DC™ protein assay (Bio-Rad, Feldkirchen, Germany) following manufacturer’s instructions. The protein solution was mixed with Invitrogen™ NuPAGE™ LDS sample buffer and Invitrogen™ NuPAGE™ reducing agent (Thermo Fisher Scientific Inc., Waltham, Massachusetts, USA) and subsequently denatured for 10 min at 70 °C. After centrifugation at 13,000 rpm for 2 min at 4 °C, equivalent amounts of protein extracts (20 µg per lane) were separated by LDS-PAGE using Invitrogen™ NuPAGE™ Bis–Tris Mini Protein Gels, 10% (Thermo Fisher Scientific Inc., Waltham, Massachusetts, USA). Proteins were transferred to an activated PVDF membrane using a semi-dry system (Trans-Blot® SD Semi-Dry Transfer Cell, Bio-Rad, Hercules, CA, USA). After blotting, a total protein membrane staining was conducted using the AzureRed Fluorescent Total Protein Stain (Azure Biosystems, Dublin, CA, USA) according to the manufacturer’s protocol. Afterwards, the membrane was blocked in PBS-T containing 5% BSA for 1h at room temperature (r. t.) and was then incubated with anti-CAPN3 (ab223766, abcam, Cambridge, UK) at 4 °C overnight. The next day, the membrane was washed in PBS-T and incubated with secondary anti-rabbit POD antibody (G9295, Sigma–Aldrich, St. Louis, MO, USA) for 1 h at r. t. before washing with PBS-T. Antibody binding was detected by chemiluminescent substrate Radiance Q (AC2101, Azure Biosystems, Dublin, CA, USA) in the c600 Imaging System (Azure Biosystems, Dublin, CA, USA), as well as total protein staining. Densitometry was performed using AzureSpot Pro (Azure Biosystems, Dublin, CA, USA). Specific signals were normalized to the total amount of detectable proteins and data were expressed as relative to wildtype.

### Histological staining and morphometric analysis

Cryosections of 5 µm thickness of psoas, soleus, and gastrocnemius muscle were prepared and haematoxylin and eosin (H&E) staining was performed according to standard procedures^22^. Additionally, staining with nicotinamide adenine dinucleotide (NADH), cytochrome c oxidase (COX), succinate dehydrogenase (SDH), elastica van Gieson (EVG) and ATPase 9.6 were performed using standard procedures. For morphometric analysis, one cross section of each muscle stained with H&E was scanned using a Keyence microscope BZ-X810 (Keyence, Osaka, Japan). Two regions of interest (ROI) of the same size (0.25 mm^2^) per section were selected using Fiji (v2.9.0). Adapted to a protocol published before^23^, cellular segmentation was conducted with CellPose^24,25^ and quantification of cell parameters, including number of cells, area, Feret’s diameter and minimal Feret’s diameter, were analysed using Fiji (v2.9.0) (see Supplementary Figure S2). Cells with centralized nuclei were counted manually, and the percentage was calculated by dividing by the number of cells in the respective ROI.

### Preparation of mouse skeletal muscle tissue for transmission electron microscopy (TEM)

For TEM analysis, skeletal muscle tissue (psoas muscle) was harvested from mice immediately after dissection and fixed in 2.5% glutaraldehyde (GA) for 6 h at r. t.. The tissue was then cut into approximately 1 mm^3^ biopsy pieces and further fixed in 2.5% GA. This was followed by three 10-min washes in 0.1 M phosphate buffer (PB) at r. t.. Post-fixation was performed using 1% osmium tetroxide for 2.5 h at r. t., followed by three additional 10-min PB washes. The tissues were then dehydrated in an ascending ethanol row (50%, 70%, 2 × 80%, 90%, 96%, and 3 × 100% ethanol dried over molecular sieves). Each step lasted 15 min, except for the 70% ethanol step, which was carried out overnight at 4 °C. This step also included 1% phosphotungstic acid and 1% uranyl acetate for contrast enhancement. Prior to resin infiltration, the samples were treated with propylene oxide (PO) for 10 min. Infiltration with epoxy resin was performed gradually at r. t. in the following steps: 1:3 (epoxy:PO) for 1 h, 1:1 for 1 h, and 3:1 for 2 h. The tissues were then incubated in pure epoxy resin overnight and polymerized for 48 h. Samples were roughly trimmed to ~ 1 mm3 using a trimming device (EM Rapid, Leica). Ultrathin sections (~ 70 nm) were cut with an ultramicrotome (UC Enuity, Leica) and transferred on 75-mesh copper grids.

### TEM-imaging and image-post-processing

TEM imaging was performed using a Zeiss EM910 transmission electron microscope equipped with a tungsten cathode, operated at 80 kV and 5–7 µA, using ImageSP software (Version 1.2.13.33 (× 64), TRS). Digital 16-bit images were acquired with a TRS CCD camera at a resolution of 2048 × 2048 pixels and at various magnifications. Scale bars are included in the images. Image post-processing was carried out using Fiji (Version 2.14.0/1.54f;^26^) for scaling, brightness/contrast adjustment, and scale bar insertion. Final figure compositions were created using Inkscape (Version 1.3.2, https://inkscape.org). For quantitative analysis of sarcomere length, 10–15 TEM images per time point were randomly selected per mouse (n = 3). Multiple sarcomeres per image were manually measured using Fiji (v2.9.0).

### Statistical analysis

When suitable, a two-sided Student’s *t*-test was used. A 2-way ANOVA was carried out to test for differences between genotype and gender and interactions between genotype and gender. For comparisons over time, an ANOVA with repeated measurements was conducted, followed by a post-hoc Bonferroni test if appropriate. Statistical testing and graphics were performed with Microsoft Excel and GraphPad Prism (Version v10.4.1, GraphPad Software, San Diego, CA, USA). Data are expressed as the mean ± standard deviation (*p < 0.05, **p < 0.01, ***p < 0.001, ****p < 0.0001).

## Results

### Capn3-deficient mice demonstrate no manifest clinical phenotype until the age of 15 months

**Fig. 1:**
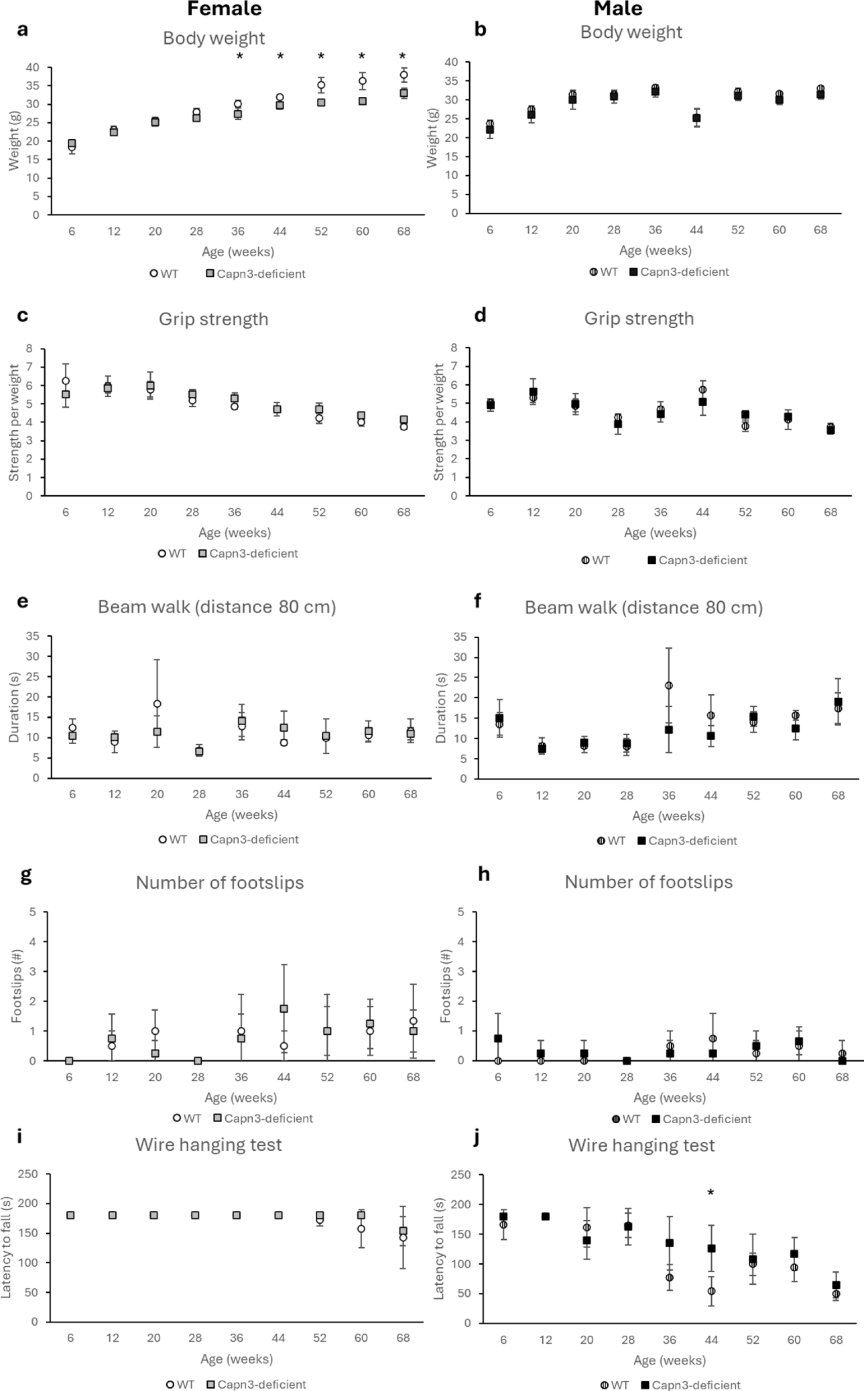
Results of in vivo phenotyping of n = 3–4 WT and n = 3–4 Capn3-deficient mice per gender are shown. (**a**) Body weight of females was significantly reduced in Capn3-deficient mice from 36 weeks of age. (**b**) In males, no differences in weight between genotypes was observed. (**c**–**h**) No differences in motor function were captured via grip strength (**c**,**d**), duration of beam walk (**e**,**f**) and number of foot slips during beam walk (**g**,**h**) between female and male WT and Capn3-deficient mice. (**i**) Female WT and Capn3-deficient mice did not present differences in latency to fall during wire hanging test. (**j**) Male WT and Capn3-deficient mice showed an impairment during ageing and WT mice performed significantly worse at the age of 44 weeks. Asterisks indicate significance comparing genotypes within timepoints.

The phenotype of Capn3-deficient mice was observed at the age of 6 weeks, 12 weeks and then every 8 weeks up to an age of 68 weeks. Female Capn3-deficient mice (Fig. 1a) showed a significantly reduced weight compared to WT mice, beginning from the age of 36 weeks ( WT 30.18 ± 0.94 g, Capn3-deficient 27.45 ± 1.62 g, *p* = 0.045) up to the age of 68 weeks (44 weeks: WT 31.95 ± 0.54 g, Capn3-deficient 29.69 ± 1.03 g, *p* = 0.015; 52 weeks: WT 35.18 ± 2.17 g, Capn3-deficient 30.41 ± 0.62 g, *p* = 0.017; 60 weeks: WT 36.37 ± 2.29 g, Capn3-deficient 30.94 ± 0.90 g, *p* = 0.015; 68 weeks: WT 37.98 ± 1.92 g, Capn3-deficient 33.03 ± 1.34 g, *p* = 0.019). However, no significant differences occurred between male WT and Capn3-deficient mice until the age of 68 weeks (*p* > 0.05) (Fig. 1b). The lower weights in males of both genotypes at the age of 44 weeks were due to a technical issue with the scale, which could not be corrected.

Male and female Capn3-deficient mice did not show significant differences in grip strength per weight compared to WT mice at any timepoint (*p* > 0.05) (Fig. 1c,d). For males, the outlier at timepoint t5 (Fig. 1d) is due to false low values of weight as mentioned before. Also, the duration needed to pass the beam walk and the number of footslips did not differ between male and female Capn3-deficient and WT mice during the observed period (*p* > 0.05) (Fig. 1e–h). The four limb wire hanging test did not show differences in latency to fall between female WT and Capn3-deficient mice with both genotypes showing a slight, non-significant impairment at higher age (F(2, 10) = 1.3906, *p* = 0.29318) (Fig. 1i). Males of both genotypes also showed an impairment during ageing (effect for time: F(6, 24) = 18.835, *p* < 0.00001, post-hoc Bonferroni 20 vs. 36 weeks *p* = 0.029, 20 vs. 44 weeks *p* = 0.00075, 20 vs. 52 weeks *p* = 0.015, 20 vs. 60 weeks *p* = 0.0499, 20 vs. 68 weeks *p* < 0.00001) (Fig. 1j). At the age of 44 weeks, WT mice performed significantly worse compared to Capn3-deficient mice (WT 54.25 ± 24.30 s vs. Capn3-deficient 126.25 ± 38.78 s, *p* = 0.034).

### The Capn3-deficient mouse model shows similar transcription levels as WT, whereas translation of CAPN3 is almost completely prevented

**Fig. 2:**
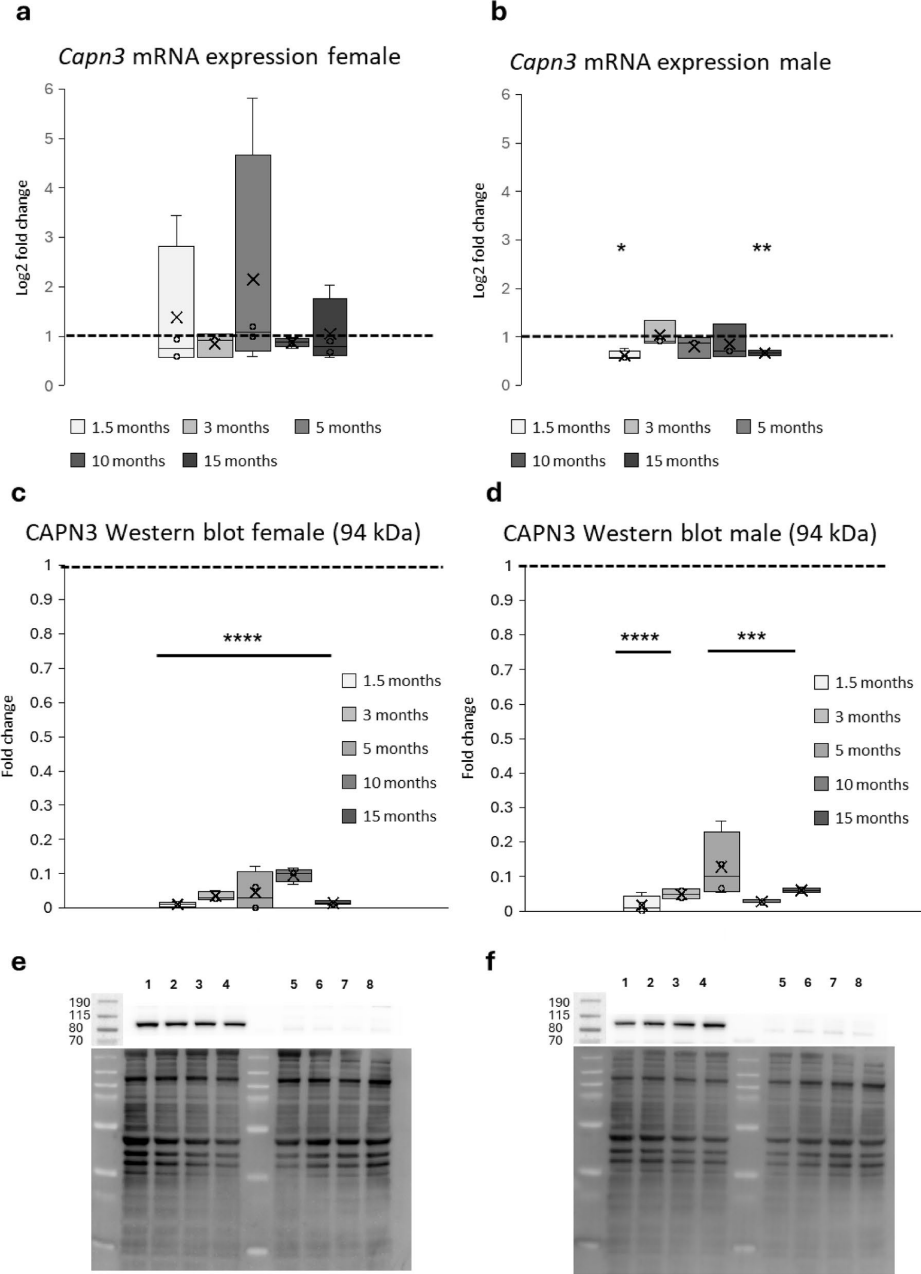
Analysis of *Capn3*-mRNA expression and CAPN3 western blot in WT and Capn3-deficient mice. (**a**) No significant differences in expression of *Capn3*-mRNA between female WT and Capn3-deficient mice were detected at any timepoint, but Capn3-deficient mice showed a high variance in mRNA expression. (**b**) Male Capn3-deficient mice showed significantly decreased expression of *Capn3*-mRNA at an age of 1.5 months (*p* = 0.028) and 15 months (*p* = 0.006). (**c**,**d**) The expression of whole-length 94 kDa CAPN3 protein was significantly reduced in both, female and male Capn3-deficient mice during the disease. (**e**,**f**) Examples of CAPN3 western blot of gastrocnemius and soleus muscles of female (**e**) and male (**f**) WT (sample 1–4) and Capn3-deficient (sample 5–8) mice at the age of 3 months. Asterisks indicate significance comparing genotypes within timepoints. See Supplementary Figure S3 for full-length western blots.

To assess the molecular effects of the 1759 nucleotide deletion mutation by CRISPR/Cas9 endonuclease-mediated genome editing, real-time qPCR was carried out to quantify the levels of *Capn3*-mRNA within the soleus and gastrocnemius muscles during the disease. Primers have been designed to bind to a sequence that spans exons 4 and 5 of the *Capn3* gene. In females, no significant differences in the amount of *Capn3*-mRNA between WT and Capn3-deficient mice have been detected at any timepoint, but Capn3-deficient mice showed a high variance in mRNA expression (Fig. 2a). Male Capn3-deficient mice showed significantly decreased expression of *Capn3*-mRNA (Fig. 2b) at an age of 1.5 months (*p* = 0.028) and 15 months (*p* = 0.006). However, the expression of the full-length 94 kDa CAPN3 protein was significantly reduced to a minimal level in both female and male Capn3-deficient mice during the disease (fold change female: 1.5 months 0.01 ± 0.007; 3 months 0.03 ± 0.01; 5 months 0.05 ± 0.05; 10 months 0.10 ± 0.02; 15 months 0.02 ± 0.005, all *p* < 0.0001, fold change male: 1.5 months 0.02 ± 0.02, *p* < 0.0001; 3 months 0.05 ± 0.01, *p* < 0.0001; 5 months 0.13 ± 0.08, *p* = 0.0007; 10 months 0.03 ± 0.003, *p* = 0.0001; 15 months 0.06 ± 0.006, *p* = 0.0009) (Fig. 2c–f) (see Supplementary Figure S3 for uncropped western blots).

### Morphometric analysis of gastrocnemius myocytes as a slightly affected muscle shows an affection in Capn3-deficient mice at higher ages

**Fig. 3:**
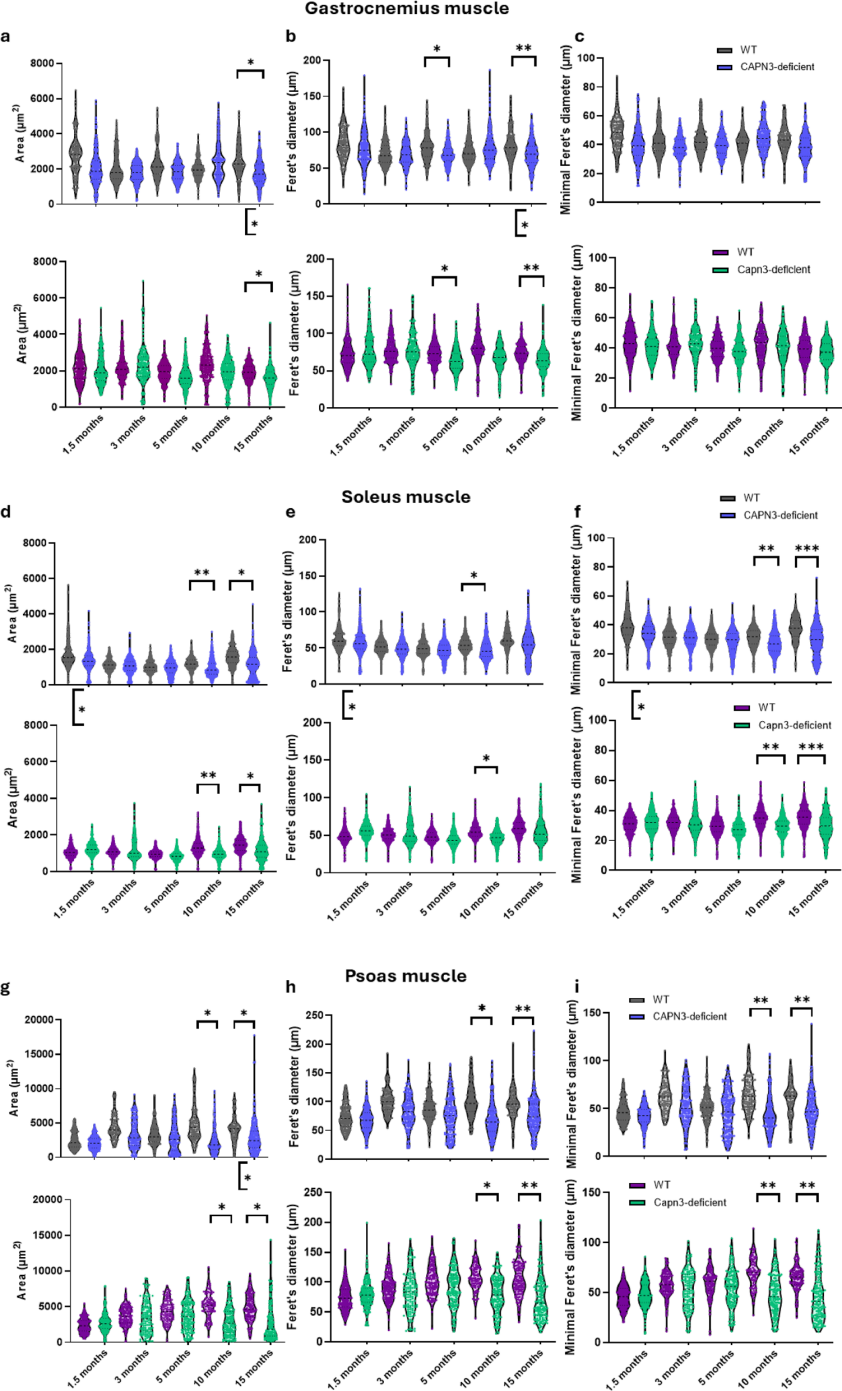
Cell size was measured as area, Feret’s diameter and minimal Feret’s diameter in gastrocnemius, soleus and psoas muscles. (**a**) Area of myocytes of gastrocnemius muscle in female (top) and male (bottom) Capn3-deficient mice was reduced at the age of 15 months compared to WT, and area was smaller in males than in females. (**b**) Feret’s diameter of gastrocnemius myocytes in female (top) and male (bottom) Capn3-deficient mice was reduced at the age of 5 and 15 months compared to WT, and Feret’s diameter was smaller in males than in females at the age of 15 months. (**c**) Minimal Feret’s diameter of gastrocnemius myocytes did not show gender- or genotype-specific differences (female: top, male: bottom). (**d**) In soleus muscle, area of myocytes of males (bottom) was significantly smaller compared to females (top) at the age of 1.5 months. The area of myocytes of Capn3-deficient mice was significantly reduced at the age of 10 and 15 months, regardless of gender. (**e**) In soleus muscle, Feret’s diameter of myocytes of males (bottom) was significantly smaller compared to females (top) at the age of 1.5 months. Feret’s diameter of myocytes of Capn3-deficient mice was significantly reduced at the age of 10 months, regardless of gender. (**f**) In soleus muscle, minimal Feret’s diameter of myocytes of males (bottom) was significantly smaller compared to females (top) at the age of 1.5 months. Minimal Feret’s diameter of myocytes of Capn3-deficient mice was significantly reduced at the age of 10 and 15 months, regardless of gender. (**g**) In psoas muscle, the area of myocytes of Capn3-deficient mice was significantly reduced at the age of 10 and 15 months compared to WT, with males (bottom) presenting a reduced area compared to females (top) at the age of 15 months. (**h**,**i**) In psoas muscle, Feret’s diameter (**h**) and minimal Feret’s diameter (**i**) of myocytes of Capn3-deficient mice were significantly reduced at the age of 10 months, regardless of gender (female: top, male: bottom).

For morphometric analysis of cell size, a 2-way ANOVA was carried out for area, Feret’s diameter and minimal Feret’s diameter to test for differences between genotype and gender and interactions between genotype and gender, respectively. In gastrocnemius muscle, area was significantly reduced in Capn3-deficient mice at the age of 15 months (F (1, 10) = 6.958, *p* = 0.0248). Additionally, at the age of 15 months, area was significantly different between female (Fig. 3a, top) and male (Fig. 3a, bottom) mice (F (1, 10) = 5.439, *p* = 0.0419). Importantly, there was no interaction between the parameters genotype and gender (F (1, 10) = 1.065, *p* = 0.33) (Fig. 3a; Table 1). Feret’s diameter was also significantly reduced at the age of 15 months (F (1, 10) = 12.00, *p* = 0.0061) in Capn3-deficient compared to WT mice. Again, there was a significant difference between genders (F (1, 10) = 5.754, *p* = 0.0374) with male mice (Fig. 3b, bottom) showing significantly smaller Feret’s diameter (Table 1) than females (Fig. 3b, top). No interaction between the parameters gender and genotype occurred (F (1, 10) = 0.1110, *p* = 0.7459). Additionally, at 5 months of age, Feret’s diameter was significantly reduced in myocytes of Capn3-deficient compared to WT mice (F (1, 12) = 5.120, *p* = 0.043). Analysis of minimal Feret’s diameter of myocytes in gastrocnemius muscle revealed no differences between different genotypes and genders at any time point (Fig. 3c; Table 1).

**Table 1:** Comparison of differences between Capn3-deficient and WT mice, as well as between male and female mice of area, Feret’s diameter, minimal Feret’s diameter and percentage of cells with centralized nuclei of myocytes in gastrocnemius, soleus and psoas muscle at different ages is presented.

			1.5 months			3 months		
			WT or female	Capn3-deficient or male	2-way ANOVA	WT or female	Capn3-deficient or male	2-way ANOVA
Gastrocnemius muscle	Area	Genotype	2497 ± 525.1	2182 ± 543.5	F (1, 11) = 1.629, *p* = 0.23	2275 ± 619.0	2112 ± 628.5	F (1, 11) = 0.1250, *p* = 0.73
		Gender	2456 ± 566.2	2218 ± 528.9	F (1, 11) = 1.036, *p* = 0.33	2056 ± 635.1	2363 ± 574.0	F (1, 11) = 1.003, *p* = 0.34
		Interaction			F (1, 11) = 1.044, *p* = 0.33			F (1, 11) = 1.221, *p* = 0.29
	Feret’s diameter	Genotype	78.94 ± 10.36	77.56 ± 12.42	F (1, 11) = 0.1286, *p* = 0.73	77.48 ± 11.29	74.88 ± 12.84	F (1, 11) = 0.0787, *p* = 0.78
		Gender	81.53 ± 9.109	75.29 ± 12.47	F (1, 11) = 1.220, *p* = 0.29	72.35 ± 10.24	80.74 ± 12.31	F (1, 11) = 1.848, *p* = 0.2
		Interaction			F (1, 11) = 0.5307, *p* = 0.48			F (1, 11) = 0.2712, *p* = 0.61
	Minimal Feret’s diameter	Genotype	46.06 ± 4.220	41.81 ± 5.249	F (1, 11) = 3.090, *p* = 0.11	43.48 ± 6.543	41.22 ± 4.830	F (1, 11) = 0.3826, *p* = 0.55
		Gender	44.14 ± 5.623	43.49 ± 5.015	F (1, 11) = 0.2059, *p* = 0.66	41.91 ± 6.986	43.02 ± 4.331	F (1, 11) = 0.1819, *p* = 0.68
		Interaction			F (1, 11) = 1.073, *p* = 0.32			F (1, 11) = 2.065, *p* = 0.18
	Central nuclei	Genotype	0.02168 ± 0.02474	0.01570 ± 0.03507	F (1, 26) = 0.2946, *p* = 0.59	0.007911 ± 0.01729	0.03347 ± 0.04007	**F (1, 26) = 10.47, *p* = 0.0033**
		Gender	0.02450 ± 0.03995	0.01323 ± 0.01821	F (1, 26) = 0.9542, *p* = 0.34	0.007667 ± 0.01425	0.03375 ± 0.04124	**F (1, 26) = 10.82, *p* = 0.0029**
		Interaction			F (1, 26) = 0.5372, *p* = 0.47			**F (1, 26) = 6.220, *p* = 0.0193**
Soleus muscle	Area	Genotype	1417 ± 525.3	1323 ± 222.8	F (1, 11) = 0.9223, *p* = 0.36	1119 ± 189.5	1176 ± 318.2	F (1, 11) = 0.3266, *p* = 0.58
		Gender	1583 ± 419.8	1178 ± 228.2	**F (1, 11) = 8.007, *p* = 0.0164**	1104 ± 215.3	1193 ± 293.6	F (1, 11) = 0.6274, *p* = 0.45
		Interaction			F (1, 11) = 4.324, *p* = 0.06			F (1, 11) = 1.502, *p* = 0.25
	Feret’s diameter	Genotype	56.38 ± 10.08	57.68 ± 6.214	F (1, 11) = 0.005412, *p* = 0.94	51.64 ± 3.785	52.96 ± 9.384	F (1, 11) = 0.3184, *p* = 0.58
		Gender	61.45 ± 8.577	53.25 ± 5.258	**F (1, 11) = 6.038, *p* = 0.0318**	50.75 ± 4.812	53.98 ± 8.508	F (1, 11) = 1.157, *p* = 0.31
		Interaction			F (1, 11) = 2.943, *p* = 0.11			F (1, 11) = 2.205, *p* = 0.17
	Minimal Feret’s diameter	Genotype	35.26 ± 6.272	33.08 ± 3.157	F (1, 11) = 1.943, *p* = 0.19	31.70 ± 2.808	31.51 ± 2.985	F (1, 11) = 0.001794, *p* = 0.97
		Gender	36.60 ± 5.260	31.91 ± 3.285	**F (1, 11) = 6.494, *p* = 0.0271**	31.23 ± 3.132	32.04 ± 2.505	F (1, 11) = 0.2993; *p* = 0.6
		Interaction			F (1, 11) = 3.395, *p* = 0.09			F (1, 11) = 0.3039, *p* = 0.59
	Central nuclei	Genotype	0.01315 ± 0.01594	0.009549 ± 0.01103	F (1, 26) = 1.142, *p* = 0.3	0.007642 ± 0.009844	0.08989 ± 0.1168	**F (1, 26) = 9.888, *p* = 0.0041**
		Gender	0.01844 ± 0.01551	0.004918 ± 0.006987	**F (1, 26) = 10.12, *p* = 0.0038**	0.03043 ± 0.05271	0.06384 ± 0.1174	F (1, 26) = 2.327, *p* = 0.14
		Interaction			F (1, 26) = 0.08909, *p* = 0.77			F (1, 26) = 2.570, *p* = 0.12
Psoas muscle	Area	Genotype	2649 ± 731.9	2554 ± 651.1	F (1, 11) = 0.1108, *p* = 0.75	4221 ± 1070	3528 ± 678.0	F (1, 10) = 2.117, *p* = 0.18
		Gender	2481 ± 761.0	2701 ± 604.8	F (1, 11) = 0.2462, * p* = 0.63	3978 ± 1094	3771 ± 814.0	F (1, 10) = 0.3805, * p* = 0.55
		Interaction			F (1, 11) = 1.980, * p* = 0.19			F (1, 10) = 0.5522, * p* = 0.47
	Feret’s diameter	Genotype	77.84 ± 12.65	75.48 ± 10.90	F (1, 11) = 0.1844, * p* = 0.68	98.23 ± 11.98	85.30 ± 10.70	F (1, 10) = 4.232, * p* = 0.07
		Gender	73.99 ± 13.12	78.85 ± 9.948	F (1, 11) = 0.4623, * p* = 0.51	92.27 ± 11.54	91.26 ± 14.89	F (1, 10) = 0.2029, * p* = 0.66
		Interaction			F (1, 11) = 1.563, * p* = 0.24			F (1, 10) = 0.5154, * p* = 0.49
	Minimal Feret’s diameter	Genotype	48.30 ± 6.539	46.18 ± 5.413	F (1, 11) = 0.5805, * p* = 0.46	61.46 ± 8.548	54.16 ± 6.400	F (1, 10) = 3.167, * p* = 0.11
		Gender	46.27 ± 7.125	47.96 ± 4.830	F (1, 11) = 0.1552, * p* = 0.7	58.70 ± 10.21	56.92 ± 6.236	F (1, 10) = 0.4408, * p* = 0.52
		Interaction			F (1, 11) = 1.966, *p* = 0.19			F (1, 10) = 0.2044, * p* = 0.66
	Central nuclei	Genotype	0.005767 ± 0.01214	0.08506 ± 0.1056	**F (1, 26) = 7.510, *p* = 0.0109**	0.008009 ± 0.02078	0.2446 ± 0.1184	**F (1, 24) = 60.27, *p* < 0.0001**
		Gender	0.03809 ± 0.08121	0.05677 ± 0.09228	F (1, 26) = 0.6065, * p* = 0.44	0.1189 ± 0.1386	0.1338 ± 0.1590	F (1, 24) = 2.512, * p* = 0.13
		Interaction			F (1, 26) = 0.8251, * p* = 0.37			F (1, 24) = 1.296, * p* = 0.27

			5 months			10 months		
			WT or female	Capn3-deficient or male	2-way ANOVA	WT or female	Capn3-deficient or male	2-way ANOVA
Gastrocnemius muscle	Area	Genotype	2220 ± 607.9	1821 ± 343.1	F (1, 12) = 2.693, * p* = 0.13	2267 ± 570.3	2309 ± 582.5	F (1, 11) = 7.819e-005, * p* = 0.99
		Gender	2192 ± 644.5	1849 ± 309.8	F (1, 12) = 1.983, * p* = 0.18	2299 ± 528.5	2272 ± 627.4	F (1, 11) = 0.0574, * p* = 0.81
		Interaction			F (1, 12) = 0.3922, * p* = 0.54			F (1, 11) = 3.974, * p* = 0.07
	Feret’s diameter	Genotype	77.32 ± 8.776	67.81 ± 8.032	**F (1, 12) = 5.120, *p* = 0.043**	78.56 ± 11.44	75.70 ± 11.56	F (1, 11) = 0.4597, * p* = 0.51
		Gender	75.53 ± 10.55	69.60 ± 7.858	F (1, 12) = 1.989, * p* = 0.18	77.20 ± 10.17	77.26 ± 13.07	F (1, 11) = 0.0344, * p* = 0.86
		Interaction			F (1, 12) = 0.02785, * p* = 0.87			F (1, 11) = 4.268, * p* = 0.06
	Minimal Feret’s diameter	Genotype	42.48 ± 6.027	39.39 ± 3.361	F (1, 12) = 1.605, * p* = 0.23	42.55 ± 4.682	44.21 ± 4.652	F (1, 11) = 0.3204, * p* = 0.58
		Gender	42.53 ± 6.232	39.35 ± 2.908	F (1, 12) = 1.700, * p* = 0.22	43.56 ± 4.180	43.06 ± 5.328	F (1, 11) = 0.08736, * p* = 0.77
		Interaction			F (1, 12) = 0.3146, * p* = 0.59			F (1, 11) = 2.841, * p* = 0.12
	Central nuclei	Genotype	0.01593 ± 0.02070	0.02458 ± 0.03047	F (1, 28) = 1.033, * p* = 0.32	0.02981 ± 0.05312	0.08141 ± 0.1091	**F (1, 26) = 4.573, *p* = 0.0421**
		Gender	0.01137 ± 0.01410	0.02914 ± 0.03209	**F (1, 28) = 4.352, *p* = 0.0462**	0.02207 ± 0.03264	0.09026 ± 0.1131	**F (1, 26) = 7.259, *p *= 0.0122**
		Interaction			F (1, 28) = 2.719, *p* = 0.11			F (1, 26) = 1.655, *p* = 0.21
Soleus muscle	Area	Genotype	1003 ± 174.9	892.4 ± 126.8	F (1, 12) = 2.160, *p* = 0.17	1253 ± 128.4	987.5 ± 186.3	**F (1, 11) = 11.22, *p* = 0.0065**
		Gender	1006 ± 195.3	889.7 ± 88.58	F (1, 12) = 2.373, *p* = 0.15	1089 ± 190.6	1175 ± 225.2	F (1, 11) = 0.5505, *p* = 0.47
		Interaction			F (1, 12) = 0.02650, *p* = 0.87			F (1, 11) = 1.862, *p* = 0.2
	Feret’s diameter	Genotype	49.42 ± 4.438	45.60 ± 3.074	F (1, 12) = 3.833, *p* = 0.07	54.52 ± 2.500	48.45 ± 5.232	**F (1, 11) = 8.220, *p* = 0.0153**
		Gender	48.65 ± 5.216	46.38 ± 2.707	F (1, 12) = 1.357, *p* = 0.27	51.66 ± 5.300	51.71 ± 4.978	F (1, 11) = 0.06033, * p* = 0.81
		Interaction			F (1, 12) = 0.03386, * p* = 0.86			F (1, 11) = 0.8403, * p* = 0.38
	Minimal Feret’s diameter	Genotype	29.87 ± 2.298	28.01 ± 1.931	F (1, 12) = 2.967, * p* = 0.11	33.25 ± 2.653	28.82 ± 2.578	**F (1, 11) = 13.89, *p* = 0.0033**
		Gender	29.59 ± 2.764	28.30 ± 1.547	F (1, 12) = 1.433, * p* = 0.25	29.81 ± 2.886	32.74 ± 3.468	F (1, 11) = 4.607, * p* = 0.055
		Interaction			F (1, 12) = 0.07462, * p* = 0.79			F (1, 11) = 1.604, * p* = 0.23
	Central nuclei	Genotype	0.01200 ± 0.02526	0.1204 ± 0.08926	**F (1, 28) = 20.79, *p* < 0.0001**	0.02629 ± 0.05243	0.1652 ± 0.1526	**F (1, 26) = 12.71, *p* = 0.0014**
		Gender	0.07480 ± 0.08504	0.05759 ± 0.08650	F (1, 28) = 0.5243, *p* = 0.48	0.1418 ± 0.1550	0.03317 ± 0.05497	**F (1, 26) = 8.448, *p* = 0.0074**
		Interaction			F (1, 28) = 0.03181, * p* = 0.86			F (1, 26) = 2.740, * p* = 0.11
Psoas muscle	Area	Genotype	3919 ± 933.6	3515 ± 859.3	F (1, 12) = 0.9524, * p* = 0.35	4689 ± 828.9	3097 ± 1199	**F (1, 10) = 7.438, *p* = 0.0213**
		Gender	3302 ± 845.5	4132 ± 771.7	F (1, 12) = 4.014, * p* = 0.06	3781 ± 1437	4004 ± 1229	F (1, 10) = 5.823e-005, * p* = 0.99
		Interaction			F (1, 12) = 0.4274, * p* = 0.53			F (1, 10) = 0.9112, * p* = 0.36
	Feret’s diameter	Genotype	97.08 ± 14.54	86.44 ± 12.37	F (1, 12) = 2.938, * p* = 0.11	109.8 ± 20.38	76.95 ± 14.00	**F (1, 10) = 9.926, *p* = 0.0103**
		Gender	85.24 ± 12.36	98.27 ± 13.39	F (1, 12) = 4.408, * p* = 0.0576	92.54 ± 28.66	94.43 ± 18.29	F (1, 10) = 0.03379, * p* = 0.86
		Interaction			F (1, 12) = 0.1425, * p* = 0.71			F (1, 10) = 0.06675, * p* = 0.8
	Minimal Feret’s diameter	Genotype	56.44 ± 6.550	52.70 ± 9.821	F (1, 12) = 0.8461, * p* = 0.38	71.32 ± 14.86	47.70 ± 9.484	**F (1, 10) = 10.13, *p* = 0.0098**
		Gender	51.30 ± 8.816	57.85 ± 6.717	F (1, 12) = 2.592, * p* = 0.13	59.20 ± 20.36	59.92 ± 13.51	F (1, 10) = 0.009501, * p* = 0.92
		Interaction			F (1, 12) = 0.1457, * p* = 0.71			F (1, 10) = 0.03838, * p* = 0.85
	Central nuclei	Genotype	0.01370 ± 0.02551	0.1967 ± 0.08265	**F (1, 28) = 66.91, *p* < 0.0001**	0.04103 ± 0.05051	0.2748 ± 0.08950	**F (1, 24) = 74.14, *p* < 0.0001**
		Gender	0.1043 ± 0.1056	0.1062 ± 0.1192	F (1, 28) = 0.007105, * p* = 0.93	0.1495 ± 0.1267	0.1692 ± 0.1586	F (1, 24) = 0.5029, * p* = 0.49
		Interaction			F (1, 28) = 0.00093, * p* = 0.98			F (1, 24) = 1.543, * p* = 0.23

			15 months					
			WT or female	Capn3-deficient or male	2-way ANOVA			
Gastrocnemius muscle	Area	Genotype	2165 ± 430.1	1762 ± 322.6	**F (1, 10) = 6.958, *p* = 0.0248**			
		Gender	2135 ± 519.1	1792 ± 215.0	**F (1, 10) = 5.439, *p* = 0.0419**			
		Interaction			F (1, 10) = 1.065, * p* = 0.33			
	Feret’s diameter	Genotype	77.73 ± 6.821	67.98 ± 6.476	**F (1, 10) = 12.00, *p* = 0.0061**			
		Gender	75.83 ± 9.251	69.88 ± 6.141	**F (1, 10) = 5.754, *p* = 0.0374**			
		Interaction			F (1, 10) = 0.1110, * p* = 0.75			
	Minimal Feret’s diameter	Genotype	41.69 ± 4.096	38.32 ± 3.204	F (1, 10) = 4.247, * p* = 0.067			
		Gender	41.28 ± 4.965	38.73 ± 2.282	F (1, 10) = 2.802, * p* = 0.13			
		Interaction			F (1, 10) = 1.038, * p* = 0.33			
	Central nuclei	Genotype	0.02932 ± 0.02536	0.08908 ± 0.05544	**F (1, 24) = 12.79, *p* = 0.0015**			
		Gender	0.05978 ± 0.06138	0.05863 ± 0.04326	F (1, 24) = 0.1966, * p* = 0.66			
		Interaction			F (1, 24) = 0.1562, * p* = 0.7			
Soleus muscle	Area	Genotype	1533 ± 224.3	1174 ± 208.5	**F (1, 10) = 8.891, *p* = 0.0138**			
		Gender	1360 ± 325.5	1347 ± 251.0	F (1, 10) = 0.2845, * p* = 0.61			
		Interaction			F (1, 10) = 0.4569, * p* = 0.51			
	Feret’s diameter	Genotype	60.84 ± 3.879	55.02 ± 8.206	F (1, 10) = 2.474, * p* = 0.15			
		Gender	57.84 ± 8.047	58.02 ± 6.112	F (1, 10) = 0.03170, * p* = 0.86			
		Interaction			F (1, 10) = 0.1758, * p* = 0.68			
	Minimal Feret’s diameter	Genotype	36.40 ± 2.938	29.49 ± 1.847	**F (1, 10) = 26.60, *p* = 0.0004**			
		Gender	32.75 ± 5.208	33.14 ± 3.563	F (1, 10) = 0.2060, * p* = 0.66			
		Interaction			F (1, 10) = 1.234, * p* = 0.29			
	Central nuclei	Genotype	0.03763 ± 0.04982	0.2598 ± 0.08783	**F (1, 24) = 64.76, *p* < 0.0001**			
		Gender	0.1691 ± 0.1189	0.1283 ± 0.1474	F (1, 24) = 0.1122, * p* = 0.74			
		Interaction			F (1, 24) = 1.556, * p* = 0.22			
Psoas muscle	Area	Genotype	2165 ± 430.1	1762 ± 322.6	**F (1, 10) = 6.958, *p* = 0.0248**			
		Gender	2135 ± 519.1	1792 ± 215.0	**F (1, 10) = 5.439, *p* = 0.0419**			
		Interaction			F (1, 10) = 1.065, * p* = 0.33			
	Feret’s diameter	Genotype	102.3 ± 9.992	77.10 ± 12.14	**F (1, 10) = 17.77, *p* = 0.0018**			
		Gender	87.29 ± 14.91	92.13 ± 19.55	F (1, 10) = 0.04516, * p* = 0.84			
		Interaction			F (1, 10) = 2.222, * p* = 0.17			
	Minimal Feret’s diameter	Genotype	63.78 ± 6.670	48.58 ± 9.332	**F (1, 10) = 11.30, *p* = 0.0072**			
		Gender	55.81 ± 12.14	56.56 ± 10.89	F (1, 10) = 0.1006, * p* = 0.76			
		Interaction			F (1, 10) = 0.8664, * p* = 0.37			
	Central nuclei	Genotype	0.06754 ± 0.07212	0.2797 ± 0.08263	**F (1, 24) = 63.22, *p* < 0.0001**			
		Gender	0.1607 ± 0.1225	0.1866 ± 0.1446	**F (1, 24) = 4.288, *p* = 0.0493**			
		Interaction			F (1, 24) = 1.406, *p* = 0.25			

F- and *P*-values of 2-way ANOVA are shown. Results are presented as mean ± standard deviation. Significant effects are highlighted in bold.

Additionally, quadriceps muscle and diaphragm were also dissected and examined, but showed only mild histopathological alterations at the age of 15 months (see Supplementary Figure S1).

### Morphometric analysis of soleus myocytes reveals early gender-specific differences and pathological changes from the age of 10 months in Capn3-deficient mice

Cell size of myocytes of soleus muscle was significantly smaller in male compared to female mice at an age of 12 weeks regardless of genotype (Fig. 3d–f; Table 1). This applies for cell area (F (1, 11) = 8.007, *p* = 0.0164), as well as for Feret’s diameter (F (1, 11) = 6.038, *p* = 0.0318) and minimal Feret’s diameter (F (1, 11) = 6.494, *p* = 0.0271). At an older age, no gender-specific differences in myocyte morphology of soleus muscle occurred. Cell size was significantly decreased in the soleus muscle of Capn3-deficient mice at an age of 10 months (area: F (1, 11) = 11.22, *p* = 0.0065, Feret’s diameter: F (1, 11) = 8.220, *p* = 0.0153, minimal Feret’s diameter: F (1, 11) = 13.89, *p* = 0.0033) (Fig. 3d–f; Table 1). At 15 months of age, area (F (1, 10) = 8.891, *p* = 0.0138) and minimal Feret’s diameter (F (1, 10) = 26.60, *p* = 0.0004) of myocytes of Capn3-deficient mice were still decreased compared to myocytes of WT mice (Fig. 3d,f; Table 1). However, Feret’s diameter of myocytes did not reveal differences between Capn3-deficient and WT mice at that age (F (1, 10) = 2.474, *p* = 0.15) (Fig. 3e; Table 1).

### Morphometric analysis of psoas myocytes as a severely affected muscle shows only minor gender-specific differences but obvious pathological features from the age of 10 months in Capn3-deficient mice

When comparing the morphometry of myocytes of psoas muscle (Fig. 3g–i; Table 1), a significantly reduced cell size in Capn3-deficient mice compared to WT mice was identified at the age of 10 months regarding area (F (1, 10) = 7.438, *p* = 0.0213), Feret’s diameter (F (1, 10) = 9.926, *p* = 0.0103), and minimal Feret’s diameter (F (1, 10) = 10.13, *p* = 0.0098). At the age of 15 months, differences remained or became even more significant, respectively (area: F (1, 10) = 6.958, *p* = 0.0248, Feret’s diameter: F (1, 10) = 17.77, *p* = 0.0018, minimal Feret’s diameter: F (1, 10) = 11.30, *p* = 0.0072). Furthermore, regarding distribution of cell size at the age of 15 months, the range of cell area of female WT mice spanned 9214 µm^2^, while the range of cell area of Capn3-deficient mice spanned 17662 µm^2^ with few hypertrophic and many atrophic myocytes (Fig. 3g, top). For male mice, range was 9117 µm^2^ for WT and 14245 µm^2^ for Capn3-deficient mice, respectively (Fig. 3g, bottom). Regarding gender-specific differences of cell morphology in soleus muscle, we detected only a slightly reduced cell area at the age of 15 months in male mice compared to female mice (F (1, 10) = 5.439, *p* = 0.0419) (Fig. 3g, female top, male bottom; Table 1), regardless of genotype.

### Calculation of myocytes with centralized nuclei enables quantification of muscle pathology

In two ROIs per muscle, the number of cells exhibiting centralized nuclei was counted, and the percentage of cells with centralized nuclei per total amount of myocytes per ROI was calculated (Tables 1, 2). In gastrocnemius muscle, an increased number of cells with centralized nuclei was identified in Capn3-deficient mice compared to WT at the age of 3 months (F (1, 26) = 10.47, *p* = 0.0033). However, we also detected a significant difference between female and male mice (F (1, 26) = 10.82, *p* = 0.0029) at the same timepoint, as well as a relevant interaction between parameters genotype and gender (F (1, 26) = 6.220, *p* = 0.0193). *Post-hoc* T-tests showed only genotype-specific significant differences in males (WT 1.15%, Capn3-deficient 6.34%, *p* = 0.0332), but not in females (*p* = 0.9198). Gender-specific differences were caused by differences in Capn3-deficient (*p* = 0.0086), but not in WT (*p* = 0.3637). At the age of 5 months, no genotype-specific differences were identified regarding the number of cells presenting with centralized nuclei in gastrocnemius muscle. However, again, we found a higher percentage in male than in female mice (F (1, 28) = 4.352, *p* = 0.0462). At the age of 10 months, genotypes (F (1, 26) = 4.573, *p* = 0.0421) and genders (F (1, 26) = 7.259, *p* = 0.0122) differed significantly with higher percentage of cells with centralized nuclei in males and Capn3-deficient mice, without significant interaction between parameters. At the age of 15 months, Capn3-deficient mice showed a significantly higher percentage of centralized nuclei than WT mice in gastrocnemius muscle (F (1, 24) = 12.79, *p* = 0.0015), regardless of their gender. In soleus muscle, Capn3-deficient mice presented a significantly enhanced percentage of myocytes with centralized nuclei at an age of 3 months (F (1, 26) = 9.888, *p* = 0.0041), 5 months (F (1, 28) = 20.79, *p* < 0.0001), 10 months (F (1, 26) = 12.71, *p* = 0.0014) and 15 months (F (1, 24) = 64.76, *p* < 0.0001). Gender-specific differences occurred at the age of 1.5 months (F (1, 26) = 10.12, *p* = 0.0038) and 15 months (F (1, 26) = 8.448, *p* = 0.0074), both timepoints with higher percentages in females compared to males. At none of the time points did the parameters genotype and gender interact with each other. Regarding the percentage of centralized nuclei, psoas muscle of Capn3-deficient mice showed dystrophic pathology already at the earliest examined timepoint (1.5 months of age, F (1, 26) = 7.510, *p* = 0.0109). Furthermore, the increase of percentage of centralized nuclei remained highly significant at the ages of 3 months (F (1, 24) = 60.27, *p* < 0.0001), 5 months (F (1, 28) = 66.91, *p* < 0.0001) and 10 months (F (1, 24) = 74.14, *p* < 0.0001), until the age of 15 months (F (1, 24) = 63.22, *p* < 0.0001). Gender-specific differences only occurred at the age of 15 months (F (1, 24) = 4.288, *p* = 0.0493) with male mice showing a higher percentage of centralized nuclei compared to female mice. Again, no interaction between the parameters genotype and gender could be detected.

**Table 2:** Percentage of myocytes with centralized nuclei in gastrocnemius muscle (a), soleus muscle (b) and psoas muscle (c) of male and female Capn3-deficient and WT mice at different ages and a simplified scoring system are presented. Percentages up 5% in gastrocnemius muscle until the age of 15 months, soleus and psoas muscle until the age of 10 months, and up 10% in aged soleus and psoas muscle (15 months), respectively, are classified as normal. 5–15% are classified as slightly affected, 15–25% are classified as moderately affected, > 25% are classified as severely affected.

Age	WT			Capn3-deficient			
	Female	Male	Normal	Female	Male	*p*	Score
(a) Gastrocnemius muscle							
1.5 months	2.33%	2.05%	0–5%	2.54%	0.60%	0.59	
3 months	0.43%	1.15%	0–5%	1.10%	6.34%	0.0033	5–15%
5 months	1.41%	1.78%	0–5%	0.87%	4.05%	0.32	
10 months	1.02%	4.94%	0–5%	3.39%	14.47%	0.0421	5–15%
15 months	2.12%	3.54%	0–5%	8.87%	8.96%	0.0015	5–15%
(b) Soleus muscle							
1.5 months	2.2%	0.66%	0–5%	1.59%	0.32%	0.3	
3 months	0.87%	0.65%	0–5%	5.21%	14.02%	0.0041	5–15%
5 months	1.8%	0.55%	0–5%	13.11%	10.97%	< 0.0001	5–15%
10 months	4.86%	0.39%	0–5%	23.50%	7.21%	0.0014	5–15-25%
15 months	6.25%	1.90%	0–10%	24.91%	27.41%	< 0.0001	> 25%
(c) Psoas muscle							
1.5 months	0.79%	0.42%	0–5%	6.07%	10.94%	0.0109	5–15%
3 months	0.00%	1.4%	0–5%	20.8%	29.35%	< 0.0001	15–25%
5 months	1.31%	1.43%	0–5%	19.55%	19.8%	< 0.0001	15–25%
10 months	4.74%	3.26%	0–5%	25.16%	30.58%	< 0.0001	> 25%
15 months	5.35%	7.81%	0–10%	24.1%	33.13%	< 0.0001	> 25%

To classify the severeness of muscle pathology, we developed a scoring system addressing the percentage of cells with centralized nuclei. In any muscle of male and female WT mice until the age of 10 months, the percentage of myocytes with centralized nuclei ranged between 0 and 5% (Table 2), which also counts as normal in human muscle biopsies. In soleus and psoas muscles, older male and female WT mice (15 months) presented higher percentages of centralized nuclei. Based on our histological observations, we suggest a range of 0–10% as normal for the respective age and muscle group. Gastrocnemius muscle of Capn3-deficient mice presented up to 15% cells with centralized nuclei at the ages of 3 and 10 months in males and at 15 months in males and females, respectively (Table 2). As this percentage showed significance, the percentage of 5–15% displays slight pathology of the muscle. In soleus and psoas muscles, which have been described as more affected muscles in another mouse model of calpainopathy^16^, we detected 15–20% cells with centralized nuclei at a younger age, which could be interpreted as moderate pathology. We detected more than 25% cells with centralized nuclei in soleus muscle of Capn3-deficient mice at the age of 15 months and in psoas muscle at the ages of 10 and 15 months (Table 2). This represents a severe pathology of muscle tissue.

### Other standard histopathological staining revealed no obvious differences between WT and Capn3-deficient mice

**Fig. 4:**
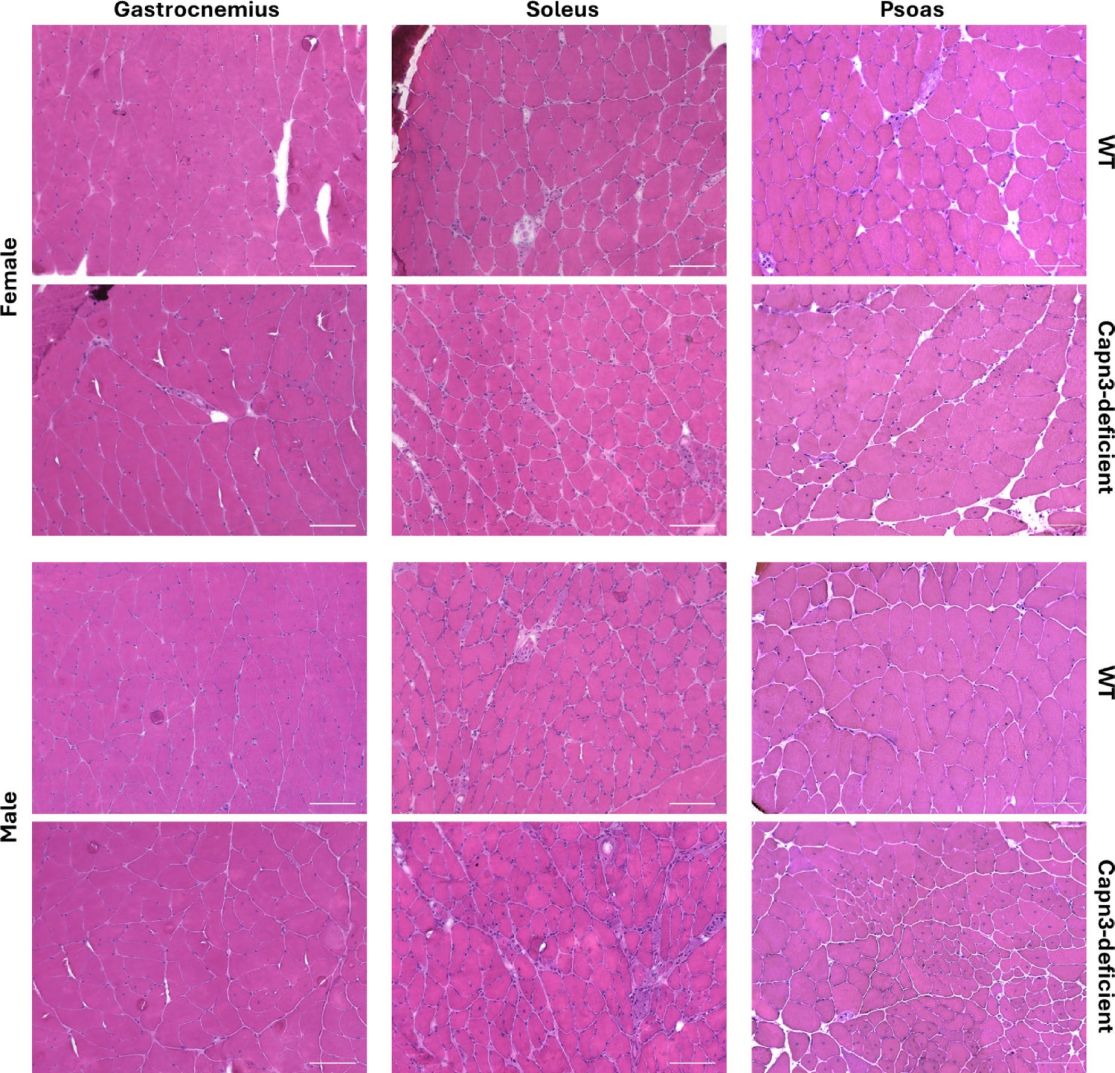
Representative examples of staining with H&E of the gastrocnemius muscle (left), soleus muscle (middle) and psoas muscle (right) of female (top) and male (bottom) wildtype (first and third row) and Capn3-deficient mice (second and fourth row) at the age of 15 months, representing the most severely affected time point. Histopathological alterations including variability of myofibre diameter and centralized nuclei are more pronounced in soleus and psoas muscle. Scale bar: 100 µm.

As already mentioned, dystrophic features such as higher variability of muscle fibre size with atrophic and hypertrophic myocytes, as well as centralized nuclei, could be detected in H&E staining (Fig. 4). Additionally, we performed other standard staining procedures, including EVG, NADH, COX/SDH, and ATPase 9.6 (see Supplementary Figure S4). Whereas EVG staining showed slightly enhanced connective tissue in Capn3-deficient mice compared to WT, other staining did not reveal differences in the normal distribution of the oxidative enzyme activity of NADH, nor in the distribution of type 1 and 2 fibres. Additionally, no COX-negative fibres could be observed in any of the muscles.

### Ultrastructural alterations in psoas muscle biopsies in Capn3-deficient mice

**Fig. 5:**
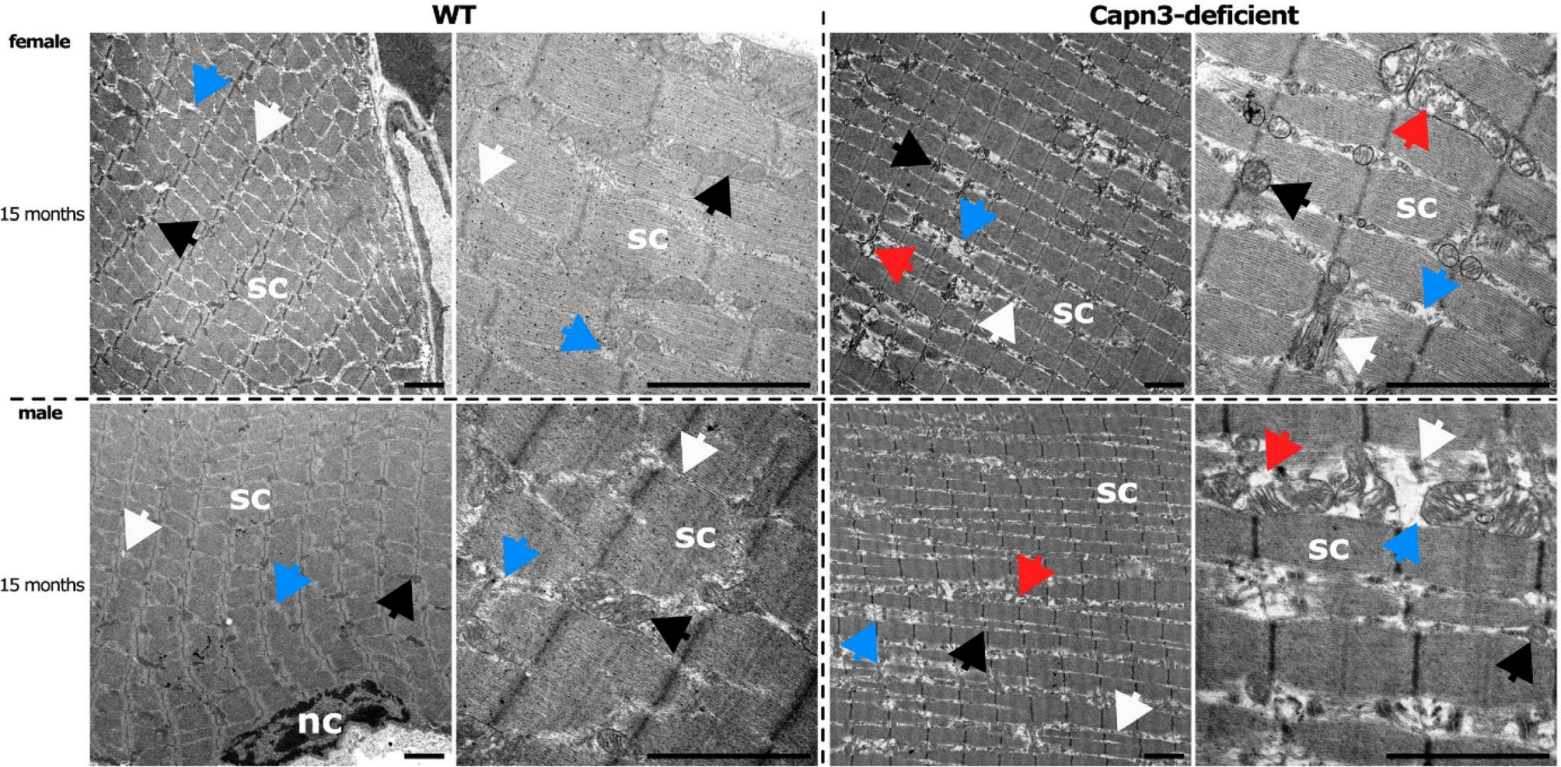
Ultrastructural analysis of psoas muscle biopsies from WT and Capn3-deficient mice using TEM. Representative TEM images of the control group (WT) depicted a physiological arrangement of sarcomeres (sc), nuclei (nc), an intact tubular system (white arrow), cytosol (blue arrow), and intact mitochondria with well-preserved cristae membrane integrity (black arrow). In contrast, the Capn3-deficient group exhibited a high variability in mitochondrial morphology, ranging from largely intact mitochondria (black arrow) to markedly altered ones. These were partially enlarged, swollen, or ruptured (red arrow). Additionally, a mild accumulation of cytosol was observed (blue arrow). The tubular system (white arrow) displayed no morphological alterations. Scale bar: 2 µm.

To validate the quantitative assessment, ultrastructural changes were further examined in detail using transmission electron microscopy (TEM). This analysis focused on the psoas muscle, as it showed the most pronounced difference in the percentage of cells with centralized nuclei between WT and Capn3-deficient mice. TEM analysis of these muscles revealed characteristic features of the ultrastructural organization (Fig. 5). Clearly visible were the regularly arranged sarcomeres (sc), nuclei (nc), an intact tubular system (white arrow), cytosol (blue arrow), and mitochondria (intact: black arrow and alterations: red arrow). No gender-specific differences were observed between female and male animals. In the WT group, the sarcomere structure, as well as the arrangement of nuclei and the tubular system, demonstrated no alterations. In contrast, the Capn3-deficient group exhibited distinct ultrastructural alterations that became more pronounced with increasing age of the animals (> 10-month-old mice). Initial mild alterations could be observed sporadically as early as 1.5 months but became increasingly manifest over time (see Supplementary Figure S5). A minimal increase in cytoplasm (blue arrow) between the myofibrils was observed (> 10-month-old mice). However, the included tubular system revealed no abnormalities. Additionally, mitochondrial alterations were more frequently observed at higher ages. The mitochondria were enlarged and, at later stages, displayed clear signs of swelling or even rupture (red arrow). The quantitative analysis of sarcomere length (see Supplementary Figure S6) revealed a tendency towards shortening in the Capn3-deficient mice observable from three months of age. Sarcomere length was significantly reduced in Capn3-deficient mice at the age of ten months (*p* = 0.0319).

These pathological alterations apparently led to a spatial displacement and loosening of the myofibrillar structure, resulting in a reduction of sarcomere length and likely affecting the contractile apparatus in Capn3-deficient mice. Overall, ultrastructural changes were observed, varying in severity and prevalence. CAPN3 deficiency led to altered cellular homeostasis, manifested particularly by mitochondrial alterations and shortened sarcomere length.

## Discussion

In this study, we described the phenotype of a new mouse model for calpainopathy in males and females until the age of 68 weeks in intervals of 8 weeks. For this purpose, we used standard tests for motor function. We detected a significantly reduced weight in female Capn3-deficient mice compared to WT mice from the age of 36 weeks up to the age of 68 weeks, while males did not differ in weight. Gender-related differences in clinical presentation of calpainopathy are also known in humans^1^. In one cohort of patients, an earlier age of onset has been described for women^27^. The same aspect was also observed in another cohort of patients in India, in which levels of creatine kinase were also higher in females^28^. Additionally, a higher amount of muscle fibre atrophy has been described for male patients with calpainopathy compared to females^29^. We next examined if motor functions were affected in this mouse model. To assess muscle strength, the grip strength of forelimbs was tested. Grip strength per body weight was not altered in Capn3-deficient mice compared to WT controls, regardless of age and gender. We also measured duration and number of foot slips during beam walk, as this test is known to be able to detect subtle deficits in motor skills and balance^30^. Again, no differences between Capn3-deficient mice and WT mice could be detected. The four limb wire hanging test has been described as a reliable and harmless method for evaluation of muscle performance in a well-known mouse model of Duchenne muscular dystrophy (mdx mice)^31^. We therefore also used the four limb wire hanging test for the mouse model of calpainopathy, again without significant differences between genotypes. At one timepoint, male WT mice performed even worse than male Capn3-deficient mice of the same age. We observed that despite training and distance from the ground, some of the male WT mice started to jump directly when placed on the grid. After noticing that jumping down is harmless, motivation to stay hanging decreased. Thus, this observation of WT performing worse than transgenics could maybe be explained by reduction of motivation or fear.

So far, three different mouse models of calpainopathy have been used in former studies^32^ (for comparison, see Table 3). One mouse model was generated by substituting exons 2 and 3 with a neoR cassette^16^, while another model is a knock-out model generated by a premature stop codon^17^. Third, a knock-in model expressing a proteolytically inactive form of CAPN3 has been generated^18^. In terms of weight, the mouse model presented in this study is comparable with the mouse model based on an inactivation of the proteolytic activity of CAPN3 due to replacements of exons 2 and 3^19^. For this mouse model, females have been described as significantly lighter than WT mice starting from 3 months of age^33^. To assess motor function in vivo, endurance treadmill, escape, and forelimb wire tests were performed in this study. A slightly reduced performance in the wire test was observed exclusively in 10-month-old transgenic mice (*p* = 0.05), while no significant differences were detected at 3 or 19 months of age^33^. For the knock-in model, a significant reduction in grip strength per body weight has been described for transgenic 20- and 32-week-old female mice^18^. In contrast to the findings in our mouse model, they also detected a significantly increased body weight in female transgenic mice at 20 and 32 weeks of age^18^. The Capn3-knock-out model was tested with a run-to-exhaustion test in two studies. 5–6-month-old male mice showed a decreased exercise performance in the run-to-exhaustion test compared to WT mice^34^, as well as 26–30-week-old and 40–44-week-old male and female transgenics, respectively^35^.

**Table 3:** Comparison of different mouse models of calpainopathy.

Study/source	Richard et al.^16^	Kramerova et al.^17^	Tagawa et al.^18^	JAX stock #031211
Mouse model	Knock-out	Knock-out	Knock-in	Capn3-deficient
Genetic Alteration	Substituting exons 2 and 3 with a neoR cassette	Premature stop codon	Expression of a proteolytically inactive form of CAPN3	1759 nucleotide deletion leading to a deletion of exon 2 and 3
Observation	3, 6, 9 months 3–19 months^33^ Female mice Male and female mice^33^	Young (2–3 months) and old (16 months) Male mice Male and female mice^35^	8, 12, 20, 32, 40, 106 weeks female mice	1.5, 3, 5, 10 and 15 months Male and female mice
Phenotypic Features	Worse performance in wire test at 10 months^33^ Lower weight in females starting at 3 months^33^	Worse performance in run-to-exhaustion test at 5–6 months (males)^34^ and 26–30 weeks and 40–44 weeks^35^	Reduction of grip strength at 20 and 32 weeks Increased weight at 20 and 32 weeks	No early clinical phenotype until 15 months Lower weight in females starting at 8 months
Key Pathological Findings	Centralized nuclei, dystrophic changes in different muscles, NF-κB signaling perturbation, IκBα accumulation	Decreased fibre area in soleus, necrosis and regeneration in different muscles, Z-disk and sarcomere defects, disorganized mitochondria	Protein aggregation, lobulated and split fibres, centralized nuclei	Decreased fibre area and perimeter, centralized nuclei, sarcomere loosening, mitochondrial damage
Therapeutic studies	Bartoli et al.^39^ Roudaut et al.^40^	Sahenk et al.^35^	No	No
Availability to scientific community	No	No	No	Yes

As no pathological motor function could be observed in this mouse model, a detailed molecular and histopathologic characterization is of high relevance for monitoring therapeutic success in drug testing studies.

Results of *Capn3*-mRNA qPCR showed an equal amount of mRNA in female WT and Capn3-deficient mice at different ages and a reduced amount in male Capn3-deficient mice only at the ages of 1.5 and 15 months, respectively. Our Western blot results proved an almost complete absence of full-length CAPN3 protein. This indicates nearly the same transcription levels of at least a fragment of *Capn3*-mRNA as in WT muscle, without indication for compensatory upregulation, and an almost complete inhibition of translation of the 94 kDa form of CAPN3. As we detected low levels of the full-length protein, the mouse model is not a true knock-out model. In fact, this might reflect the reality of patients suffering from LGMD2A/R1 better, as many mutations do not lead to an absence of the protein but a great reduction in western blot analyses^27^. The function of the remaining protein was not evaluated in this study.

As this drastic reduction of full-length CAPN3 did not have any effects on muscle function in vivo, we quantified muscle pathology in three different muscles. The gastrocnemius muscle has been described as not substantially affected, while soleus and psoas muscles were the most affected muscles in the known mouse model based on replacements of exons 2 and 3 of the *Capn3* gene ^16^. In this mouse model, pathological changes such as the presence of centralized myonuclei, area of necrosis/regeneration, splitting of fibres, and foci of mononuclear cell infiltrates have been described^16^. We focused on muscle fibre size and percentage of myocytes with centralized nuclei as quantifiable and therefore comparable data in this study, whereat inflammatory infiltrates were detected as well. Analysis of gastrocnemius muscle, assumed as non-affected, showed significantly reduced area and Feret’s diameter of myocytes in Capn3-deficient mice at the age of 15 months compared to WT mice, as well as significant differences between male and female mice. Regarding the presence of centralized nuclei, both male and female Capn3-deficient mice showed significantly more central nuclei than WT mice at the ages of 10 and 15 months. At the ages of 3, 5, and 10 months, gender-specific differences occurred with male mice presenting more centralized nuclei than females. Also, at 3 months of age, male Capn3-deficient mice showed significantly more centralized nuclei than male WT mice in soleus and psoas muscles, both described to be most affected in other mouse models^16,17^. We detected significantly reduced area, Feret’s diameter, and minimal Feret’s diameter of myocytes in male and female Capn3-deficient mice at the ages of 10 and 15 months. Again, gender-specific differences in myocyte size occurred, especially in soleus muscle at the age of 1.5 months. Regarding the pathological finding of centralized nuclei, significantly enhanced percentage of cells presenting this feature have been detected from the age of 3 months in soleus muscle and from the age of 1.5 months in psoas muscle of Capn3-deficient mice, respectively. The quantification of the percentage of cells with centralized nuclei in male and female Capn3-deficient and WT mice at different timepoints allowed us to graduate those muscles into slightly affected (gastrocnemius muscle), moderately affected (soleus muscle), and severely affected (psoas muscle). We therefore developed a scoring system to classify pathology of muscles in this mouse model regarding the percentage of myocytes with centralized nuclei, which may help to quantify and compare the severity of muscle pathology between studies using this mouse model. Histopathological scores have also been developed for human neuromuscular diseases, such as infantile Pompe disease^36^ and LGMD2A/R1^37^ and correlations to clinical severity have been observed. Importantly, as centralized nuclei can stick within the myofibres for weeks to months, the score alone should not be used for assessment of therapeutic effectiveness in drug testing studies. Another limitation of quantification of myofibre morphology is that morphological analysis of myofibres requires cross-sections cut exactly along the vertical to longitudinal axis. Especially Feret’s diameter and myofibre area can occur enlarged due to cutting angle. Therefore, analysis of Minimal Feret’s diameter is the most reliable among the ones used in this study.

As mentioned above, morphological data have also been investigated for gender-specific differences, as gender-related differences have been described for human patients suffering from LGMD2A/R1^1^. For the other mouse models of calpainopathy, either no statements about gender were made^16,38^, or only data of female^18^ or male mice^17,34,39,40^ have been presented. Recently, one study testing systemic gene transfer with recombinant AAV as a delivery vector in the knock-out model of calpainopathy also reported gender-specific differences^35^. In line with this study and the human situation, we also reported few gender-specific differences between male and female Capn3-deficient mice. In our study, more gender-specific morphologic differences occurred regardless of the genotype, as also described for humans^41,42^.

Ultrastructural descriptions of the physiological organization of sarcomeres as well as pathological features, such as mitochondrial alterations, have been extensively and comprehensively investigated and reported in muscle biopsies from male and female animals^43^. In our model, no clear ultrastructural differences between male and female animals were detected, whereas the differences between WT and Capn3-deficient mice were pronounced at higher ages. Observed alterations included mitochondrial pathologies ranging from mitochondrial swelling to mitochondrial rupture, accumulations of cytosol, and shortened sarcomeres. It should be mentioned that the changes observed might also occur as fixation artefacts. The fact that we detected these changes only in muscles of Capn3-deficient mice, although the fixation protocol was the same for all samples, and that those findings are in line with the description of disorganized and swollen mitochondria in other mouse models of calpainopathy^44^, leads to the assumption of true mitochondrial pathologies in this mouse model. Nevertheless, future mechanistic studies regarding mitochondrial pathology should confirm this finding. Furthermore, it has been proposed that loss of CAPN3 leads to structural changes in the tubular system, affecting calcium transport^45^. However, those pronounced pathologies were not confirmed in this model. The entirety of ultrastructural alterations in this model showed disrupted cellular homeostasis due to the CAPN3 deficiency, manifesting progressively as disorganization and shortening of the sarcomere structures as well as mitochondrial damage.

A limitation of our study is the relatively small sample size used for the in vivo assessments of muscle function. For morphometric investigations, sample size was sufficient to detect significant pathological changes in Capn3-deficient muscles. We described no obvious clinical phenotype of the mouse model, but we cannot exclude that small, insignificant differences we detected could be observed and significantly altered with a clearly higher sample size. Furthermore, although we carefully selected motor function tests, these tests are highly variable and prone to confounders. It might be possible that Capn3-deficient mice present abnormalities in other tests, such as endurance treadmill or run-to-exhaustion. Further studies using this mouse model should carefully consider using those or other tests for motor function. Ex vivo contraction analysis can also add valuable information about muscle function in this mouse model^35^. Additionally, Capn3-deficient mice may develop restriction of muscle function at a higher age, since we only observed the mouse model until an age of 15 months. Another limitation is the restriction of the histological analysis on morphometric data, as other pathological changes have also been described in other mouse models, such as necrosis/regeneration, splitting of fibres, and foci of mononuclear cell infiltrates^16^. As the focus of the study was quantification of changes, those aspects were not addressed in this study. Additionally, this study is purely descriptive. Further studies would be interesting, focusing on mechanisms of pathophysiology in the mouse model. Especially evaluating the enzyme function of the remaining full-length CAPN3 would be reasonable to further understand pathophysiology of this mouse model.

In conclusion, in this study, we quantified muscle morphology at different timepoints and in different muscles of both genders in the mouse model of calpainopathy to monitor muscle pathology more easily and comparably. As the mouse model did not reveal any differences in phenotype and muscle function despite decreased weight in females, it should be carefully considered to use this model for treatment studies. We identified gastrocnemius muscle as slightly affected, soleus muscle as moderately affected, and psoas muscle as severely affected in the mouse model. Moreover, a score for rating severity based on the percentage of myocytes with centralized nuclei was proposed, which could economize the number of mice, muscles and timepoints needed for future studies with the same mouse model. For new therapies, such as gene therapies, we suggest starting the treatment as early as possible, as the first pathological conspicuity occurred at the age of 6 weeks in psoas muscle. In addition, observation time should be extended, because we expect restrictions on motor functions to occur at an older age. Alternatively, other in vivo or ex vivo tests of muscle function should be added, as it is of importance to have a meaningful functional primary outcome that is able to distinguish between Capn3-deficient and WT mice. We also highlighted the relevance of testing both genders in future studies, as they present differences in weight and muscle histology.

## Supplementary Information

Below is the link to the electronic supplementary material.

Supplementary Material 1

## Data Availability

Data and further information are available upon reasonable request made to the corresponding authors.
