# Supplementary material for: Age, muscle, and gender specific characterization of muscle degeneration in a mouse model of calpainopathy
Source: Sci Rep. 2025 Sep 12;15:32507. doi: 10.1038/s41598-025-17742-3 (PMC12432131; doi:10.1038/s41598-025-17742-3)
Supplement: Supplementary file 1 — Supplementary Material 1 [file 41598_2025_17742_MOESM1_ESM.pdf]

Supplementary Information

Age, Muscle, and Gender Specific Characterization of Muscle Degeneration in a Mouse Model of Calpainopathy

Nicolina Südkamp[1,2*], Jacqueline Heinen-Weiler[3], Marlena Rohm[1,2], Michaela Zaik[1,2], Nassam Daya[1,2], Anne-Katrin Güttsches[1,2], Carsten Theiss[3,4], Andreas Roos[5,6,7], Tobias Ruck[1,2], Frank Jacobsen[1,2], Lara Schlaffke[1,2], Matthias Vorgerd[1,2*]

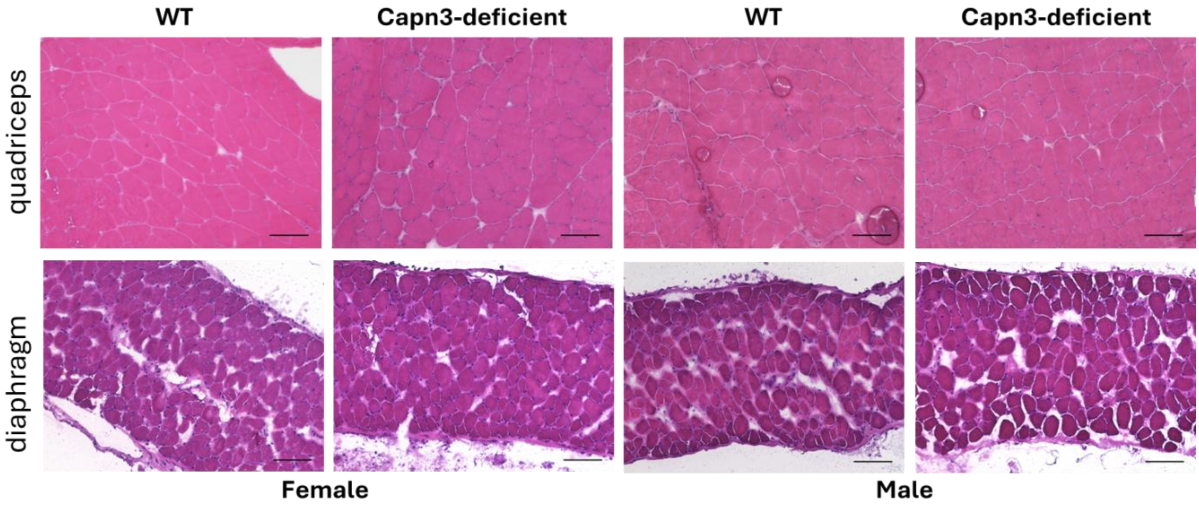

Figure S1

Representative examples of H&E staining of quadriceps muscle and diaphragm tissue of female (left) and male (right) wildtype (first and third column) and Capn3-deficient mice (second and fourth column) at the age of 15 months. Quadriceps and diaphragm showed only slight pathological features such as muscle fiber atrophy and myofibers with centralized nuclei. Scale bar: 100 µm

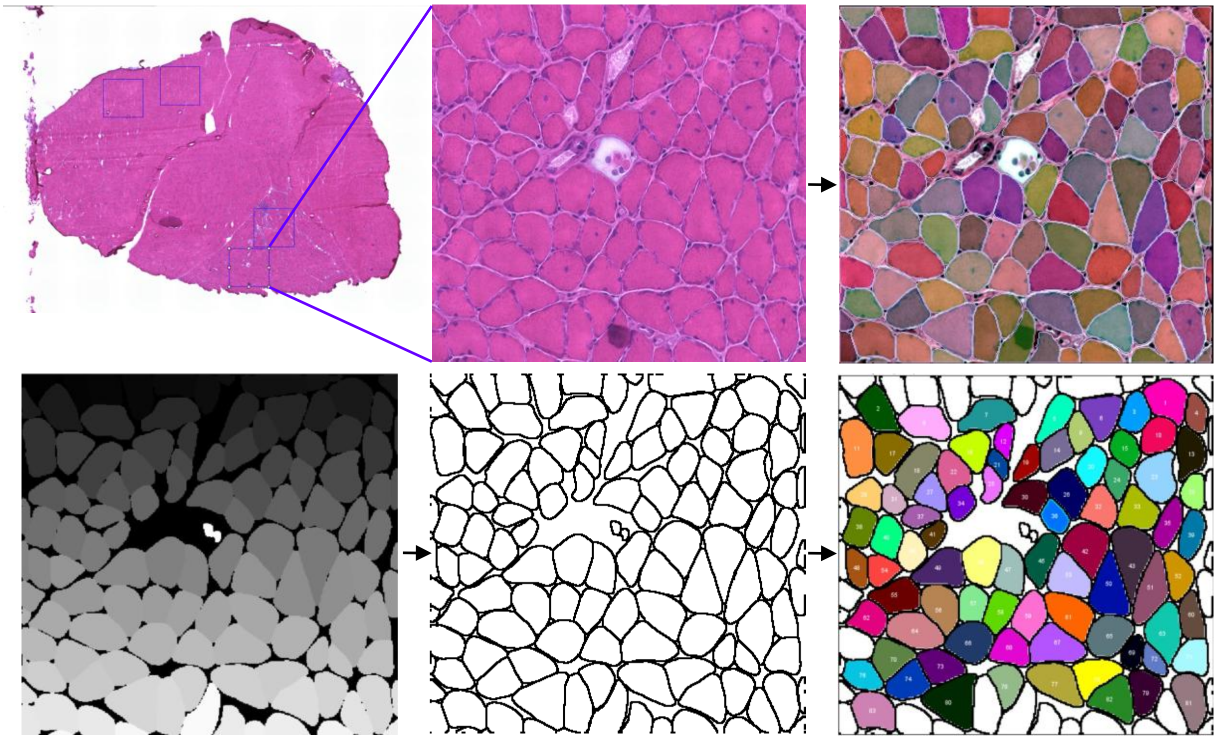

Figure S2

Two regions of interest (ROI) of the same size (0.25 mm^2) per muscle were selected using Fiji (v2.9.0). Cellular segmentation was conducted with CellPose (top). Masks were transferred to Fiji (v2.9.0) and further analyzed (bottom). Quantification of cell parameters included number of cells, area, Feret's Diameter (longest distance between two points along the selection boundary, same as maximum caliper) and minimal Feret's Diameter (minimum caliper).

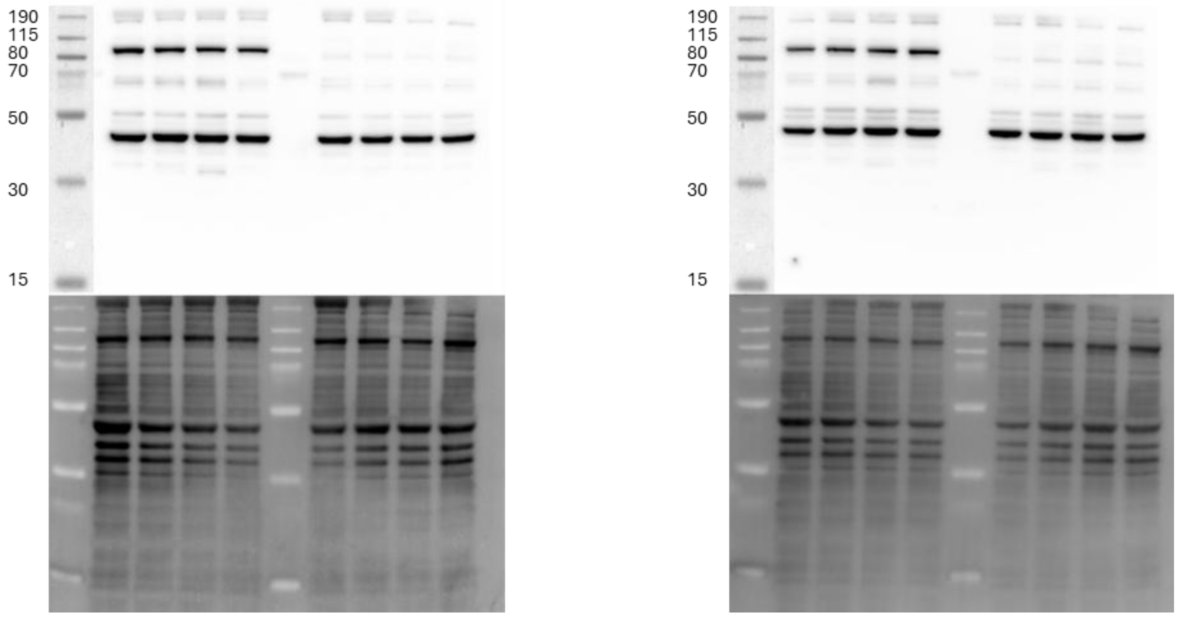

Figure S3

Uncropped CAPN3 Western Blots.

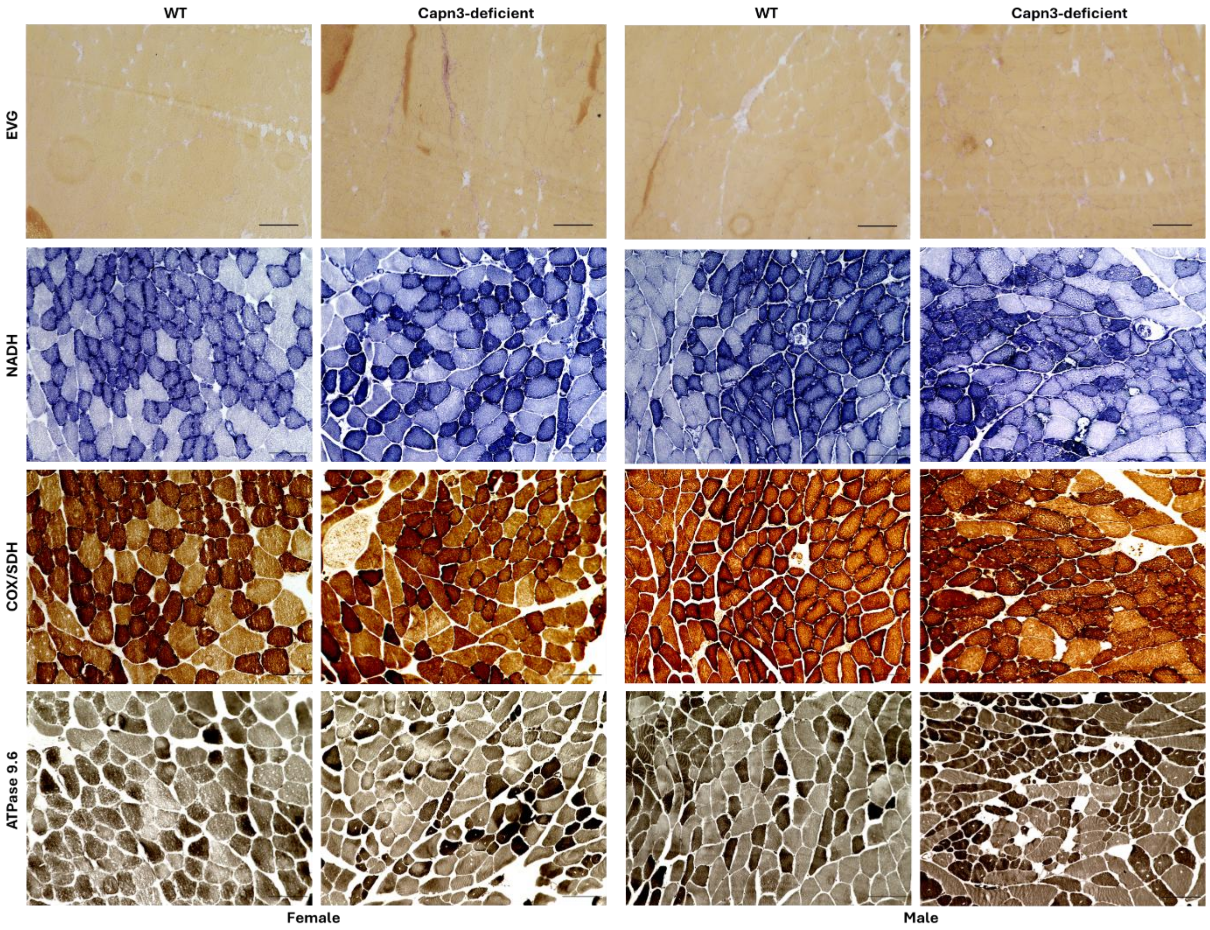

Figure S4

Staining with EVG, NADH, COX/SDH and ATPase 9.6 of the psoas muscle of female (left) and male (right) wildtype (first and third column) and Capn3-deficient mice (second and fourth column) at the age of 15 months, representing the most severely affected muscle and timepoint. Scale bar: 100 µm. Whereas EVG staining show slightly enhanced connective tissue in Capn3-deficient mice compared to WT, other staining do not reveal pathological differences.

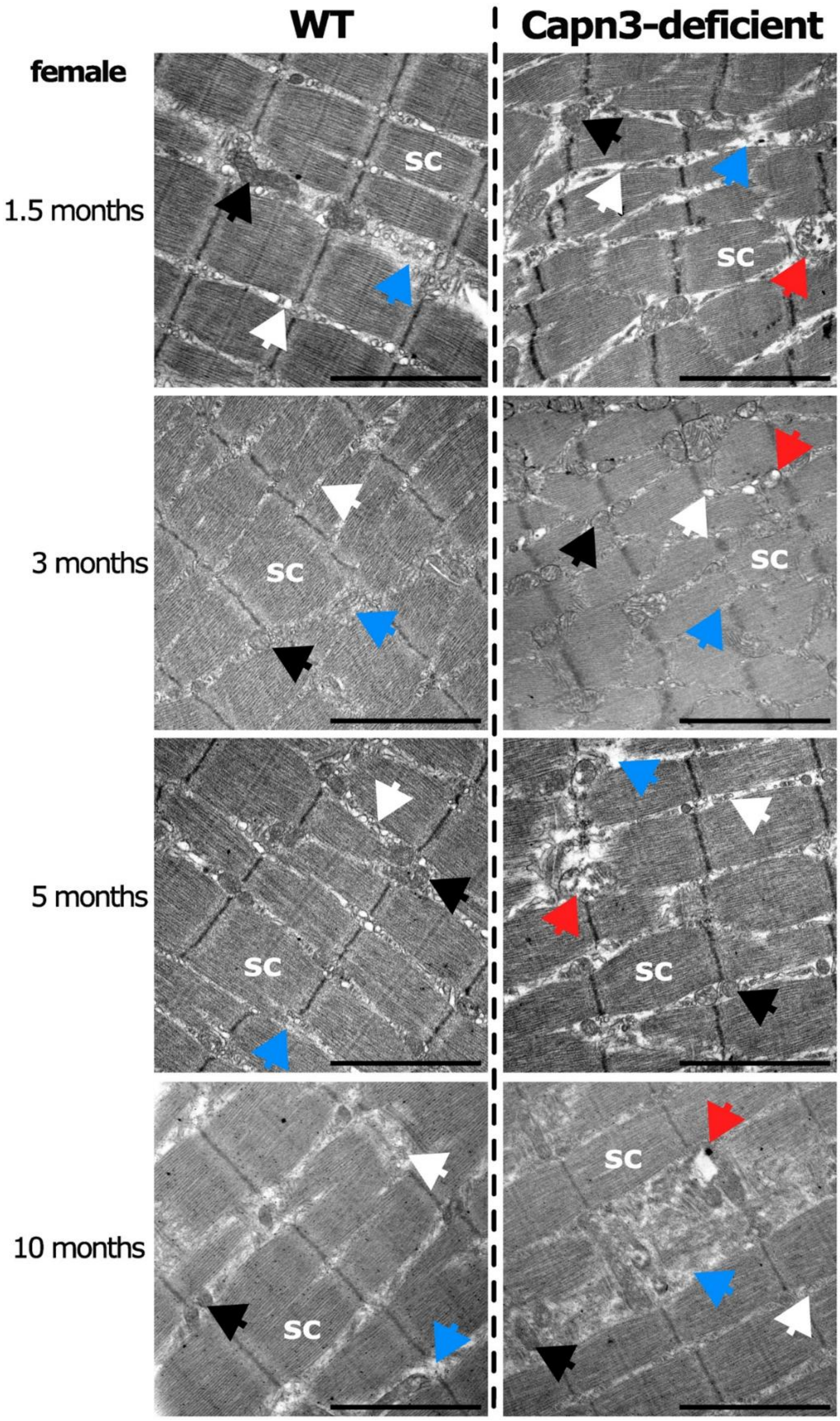

Figure S5

Ultrastructural analysis of psoas muscle biopsies from WT and Capn3-deficient mice using TEM. Representative high-magnification TEM images from the control group (WT) depict a physiological organization of sarcomeres (sc), nuclei (nc), an intact tubular system (white arrow), cytosol (blue arrow), and mitochondria with well-preserved cristae and membrane integrity (black arrow). In the Capn3-deficient mice, a pronounced variability in mitochondrial morphology was revealed, ranging from largely intact mitochondria (black arrow) to severely altered ones. These alterations included enlargement, swelling, and membrane rupture (red arrow). Additionally, a mild increase in cytosolic accumulation was revealed (blue arrow). The tubular system (white arrow) revealed no morphological alterations.

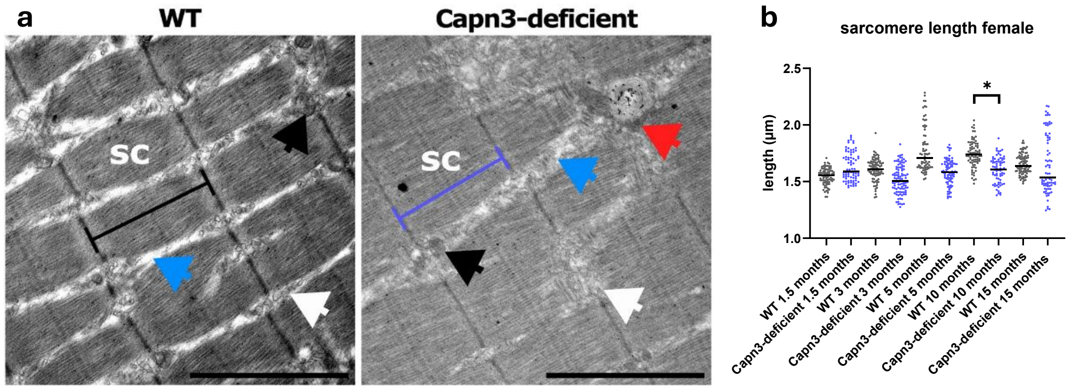

Figure S6

*(a) Ultrastructural analysis of psoas muscle biopsies from 10-month-old WT and Capn3-deficient mice using TEM. Representative high-magnification TEM images from the WT group depicted a physiological organization of sarcomeres (sc). Sarcomere lengths measured 1.65 µm (black) and 1.5 µm (blue). Additionally, the tubular system (white arrow) was intact in both groups. In contrast, the Capn3-deficient group revealed a pronounced variability in mitochondrial morphology, ranging from largely intact mitochondria with well-preserved cristae (black arrow) to altered forms. One example was a mitochondrion located close to a multilamellar body (red arrow). Additionally, a mild increase in cytosolic accumulation was revealed (blue arrow). Scale bar: 2 µm. (b) Quantitative analysis of sarcomere length of female WT and Capn3-deficient mice at 1.5, 3, 5, 10 and 15 months of age. *p<0.05.*
